# Decoding digital transformation in health professions education: a comparative analysis of knowledge, attitudes, and readiness among faculty and students at KSAU-HS

**DOI:** 10.3389/fdgth.2026.1881377

**Published:** 2026-09-15

**Authors:** Amal I. Khalil, Lamyaa Y. ALyaba, Amnah M. Jambi

**Affiliations:** 1 King Abdullah International Medical Research Center, Jeddah, Saudi Arabia; 2 College of Nursing, King Saud bin Abdulaziz University for Health Sciences, Jeddah, Saudi Arabia; 3 Ministry of National Guard Health Affairs (MNGHA), Jeddah, Saudi Arabia; 4 Psychiatric and Mental Health Nursing, Faculty of Nursing, Menoufia University, Shebin Elkom, Egypt

**Keywords:** digital competence, digital transformation, faculty, health professions education, readiness, SEM, students, technology acceptance model

## Abstract

**Introduction:**

Digital transformation is altering educational processes in health professions and needs instructors and students to acquire competencies for its implementation. At the same time, comparative information about readiness for digital transformation for educators and students in Saudi Arabian universities is scarce and the reasons for such readiness are not fully known. This study reviewed the knowledge, attitude and readiness of instructors and learners at King Saud bin Abdulaziz University for Health Sciences.

**Methods:**

The study used a cross-sectional comparative design. Data was collected from the respondents with the help of a specially designed questionnaire, based on the Technology Acceptance Model. The questionnaire measured digital competency, perceived usefulness and ease of use and facilitating conditions.

**Results:**

Respondents displayed a slightly more developed level of digital competence, perceived usefulness, perceived ease of use, and readiness for digital transformation. As for students, motivation and confidence in one's ability to work with digital technologies turned out to be the best predictors. Structural equation modeling has validated that usefulness and ease of use along with facilitating conditions represent significant factors for predicting the intention to use digital technologies. Hence, psychology and organizational aspects play a bigger role in being ready for the digital transformation than demographic variables.

**Discussion:**

The readiness for digital transformation in health professions education relates to not only the technical aspect but also to individual motivation, confidence, and institutional backing. Therefore, universities should develop specific strategies to motivate and encourage students to learn, along with training faculty, giving sufficient resources, and offering suitable organizational conditions.

## Introduction

Digital transformation in education involves the use of new technologies, such as e-learning systems, artificial intelligence (AI), and data analysis, in teaching. This shift is changing how teaching, learning, and assessment happen, especially in colleges and universities. The COVID-19 pandemic accelerated the adoption of digital tools and online learning at all levels, prompting universities to move quickly to digital teaching (1, 2). In Saudi Arabia, digital transformation in education supports Vision 2030, which calls for better digital tools and new technology to help the country develop (3).

Universities such as KSAU are increasingly using digital tools to offer blended learning, online classes, and digital materials. These changes make learning and teaching more flexible, personalized, and engaging for both teachers and students (4, 5). However, digital transformation will only work if teachers and students know how to use the new technology and are willing to adopt it.

In these environments, digital literacy is seen as essential knowledge for both educators and students. For faculty, this means being proficient with online teaching tools, such as learning management systems (LMSs), and in creating digital educational materials (6). Al Ghamdi et al. state that a key part of student learning in digital settings is possessing the necessary knowledge, including skills for using digital platforms and online resources (7). Qureshi et al. (8) add that e-learning works well only if instructors have sufficient digital skills; otherwise, targeted training is needed.

On the other hand, the shift towards digital learning in higher education presents numerous obstacles. For instance, many faculty members struggle to incorporate digital tools effectively, often due to inadequate training, limited resources, or insufficient institutional backing. Students, on the other hand, may encounter difficulties accessing and navigating online learning platforms (7, 9). The primary hurdle, however, is educators’ resistance to digital transformation, as they frequently doubt the effectiveness of online learning and fear that digital tools may undermine traditional teaching methods (10). By recognizing these challenges, institutions can better understand the factors that facilitate or hinder digital learning, enabling them to develop solutions that ease the transition process.

The success of digital transformation depends on the attitudes of both educators and students. Positive attitudes facilitate the adoption of new technology, while resistance often stems from fears of becoming outdated or of lacking sufficient support (11, 12). For example, Yan and Pourdavood (9) found that students and faculty with positive views of digital education are more likely to support it. In contrast, those with negative views may see it as a loss of traditional teaching. Al-Asiri (13) says that successful digital transformation requires not only management support but also a culture that encourages innovation. Being prepared means having digital skills, supportive environments, and a willingness to change among faculty and students (14, 15). This also requires access to resources and training, as well as strong institutional support, to overcome challenges (6, 10).

A particularly pertinent context for evaluating digital transformation readiness in KSAU-HS, one of the most distinguished health professions universities in Saudi Arabia, which has progressively integrated digital technologies into academic administration, teaching, and learning. Limited evidence exists regarding the preparedness of faculty members and students to participate in digital transformation initiatives within this context, despite ongoing investments in digital infrastructure and educational technologies. It is crucial to comprehend these viewpoints, as the successful implementation of digital innovations is contingent upon technological resources and the attitudes, competence, and willingness of users to adopt new systems. By examining both faculty and student perspectives within a single health professions institution, this study contributes to the growing body of research on digital health and educational transformation by identifying factors associated with digital transformation readiness and technology acceptance.

Research shows that students at institutions with strong digital support systems are better able to adapt to online learning (8). Faculty at these institutions also have more chances to get training and improve their digital skills (16). Assessing readiness is important for managing finances during digital transformation, as new technologies and training programs require significant investment. Institutions need to ensure these resources are used effectively. For example, Aydin (14) points out that if implementation is not planned, resources can be wasted on unused or ineffective tools. In the end, without sufficient training for faculty and students, these technologies may not yield a good return on investment (16).

Although there is an increasing focus on digital transformation in higher education, significant questions remain about how well faculty and students are equipped to embrace and use digital technologies effectively in learning environments. Earlier research has highlighted various potential obstacles that could affect digital transformation efforts, such as differences in digital skills, availability of technological resources, institutional support, and readiness to embrace new technologies (6, 10, 14, 17). Nonetheless, data on these elements in Saudi health professions education remain scarce, particularly at KSAU-HS. Gaining an understanding of the knowledge, perspectives, and preparedness of faculty and students is crucial for recognizing strengths, tackling obstacles, and guiding specific strategies to facilitate effective digital transformation. This study sought to compare faculty and students in terms of their knowledge, attitudes, and preparedness for digital transformation, and to determine the factors associated with digital transformation readiness at KSAU-HS.

## Significance of the study

Digital transformation is now seen as one of the most important parts of the future of higher education, including health professions education. Digital skills affect both learning outcomes and clinical practice, and Education 4.0 expects faculty and students to be ready to use advanced technology in teaching and learning. Many studies show that having enough knowledge, positive attitudes, and readiness to use digital tools are important for both groups (18, 19). Research also shows that digital readiness varies across groups. Student engagement depends on students’ preparedness to use technology, while faculty need strong digital confidence and sufficient institutional support to use technology effectively and teach well (20, 21). Still, there are barriers to digital transformation, such as not enough training and resistance to change (22).

In Saudi Arabia, digital transformation is a national priority, yet little research has examined the preparedness of academic communities in health sciences education for this change. This study aims to assess the readiness of faculty and students, as well as their learning environment. The findings will help institutions improve their support for effective digital transformation in health education.

## Theoretical & conceptual framework

 Figure 1 summarizes that current research is framed by established theories of technology adoption, namely the technology acceptance model and the unified theory of acceptance and use of technology. These frameworks provide a thorough understanding of how individuals form their perceptions, attitudes, and behavioral intentions towards digital technology.

**Figure 1:**
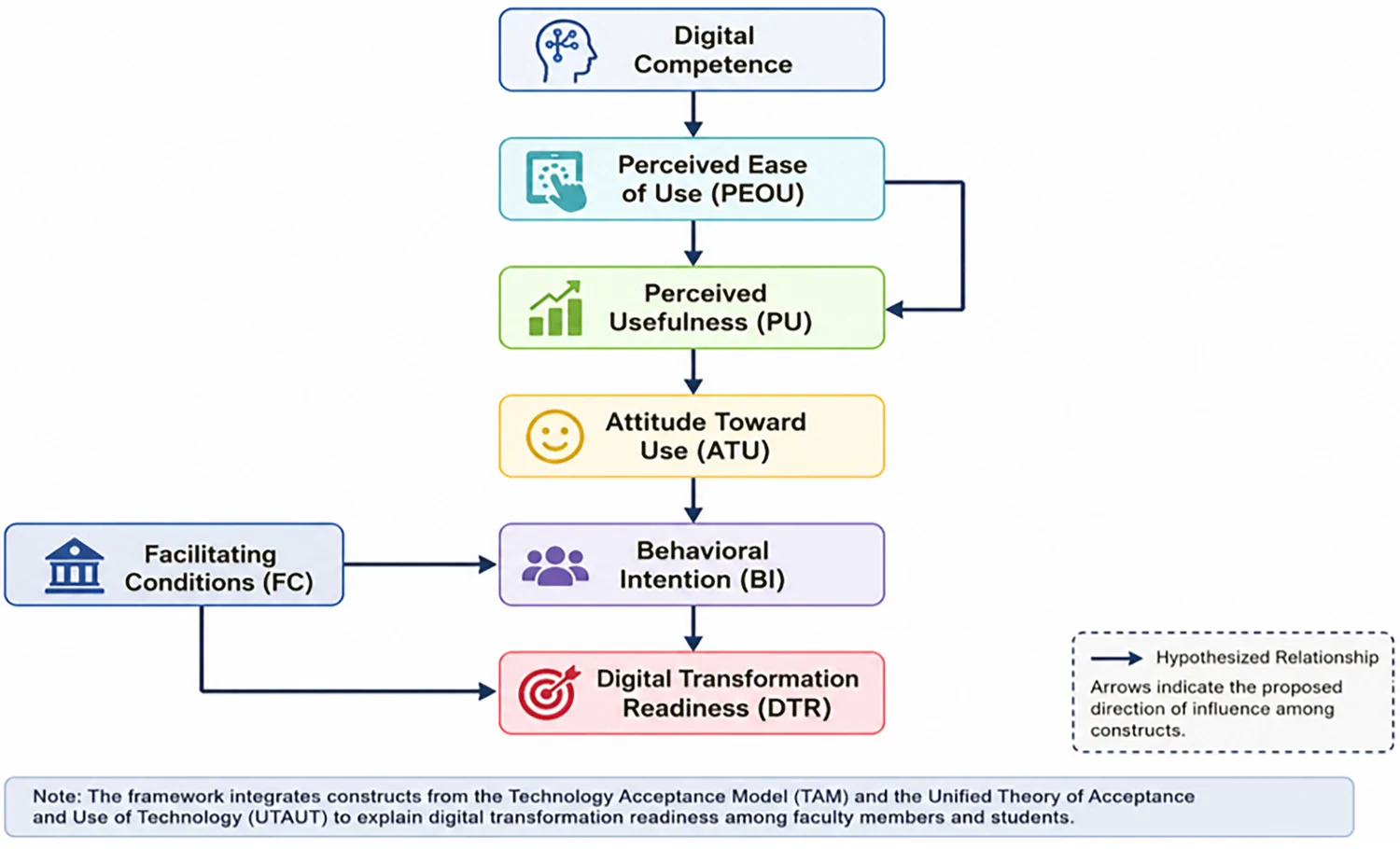
Theoretical conceptual framework based on the technology acceptance model (TAM) and unified theory of acceptance and Use of technology (UTAUT). The framework illustrates the hypothesized relationships among digital competence, perceived ease of use, perceived usefulness, facilitating conditions, attitude toward use, behavioral intention, and digital transformation readiness. The model was developed prior to data collection and guided the study hypotheses and structural equation modeling analyses.

The technology acceptance model (TAM) posits that an individual’s use of a technology is influenced primarily by perceived usefulness (PU) and perceived ease of use (PEU), which, in turn, shape attitudes and intentions (23). Digital technology will be used in this research as a foundational determinant of how an individual’s level of digital knowledge influences their perception and behavior. The higher an individual’s digital knowledge, the more likely they are to develop positive attitudes toward digital transformation, as they perceive these technologies as valuable and manageable (24).

The unified theory of acceptance and use of technology builds on previous theories by incorporating additional factors, such as facilitating conditions, social influence, and behavioral intention, into a conceptual framework explaining how one adopts a technology (24). In this framework, attitudinal response refers to an individual’s view of digital transformation. In contrast, readiness to adopt it refers to an individual’s preparedness to use digital systems in an educational institution. As a result, institutional support, training, and access to digital infrastructure are considered enabling factors that increase readiness (25). The framework was developed prior to data collection and served as the theoretical basis for the study’s hypotheses and subsequent structural equation modeling analyses. Figure 1 illustrates the hypothesized relationships among the study variables and does not present empirical findings or statistical results.

### Aim of the study

This study aimed to examine and contrast the knowledge and attitudes of faculty and students at (KSAU-HS) regarding digital transformation in education, and to determine the factors influencing their preparedness to integrate technology.

Specific Objectives

1. Assess faculty and student knowledge and attitudes regarding digital transformation.
2. Assess faculty and student attitudes toward digital technologies in educational environments.
3. Compare faculty and student perspectives on digital transformation.
4. Examine factors influencing readiness for digital transformation among faculty and students.

Hypotheses:

- **H1:** Knowledge has a significant positive effect on attitudes toward digital transformation.
- **H2:** Attitudes have a significant positive effect on readiness for digital transformation.
- **H3:** Knowledge has a direct positive effect on readiness for digital transformation.
- **H4:** Attitudes mediate the relationship between knowledge and readiness.
- **H5:** There are significant differences between faculty and students in the structural relationships among knowledge, attitudes, and readiness.

## Materials and methods

The overall methodological workflow of the study is presented in Figure 2 to provide a concise overview of the research process, from participant recruitment and data collection to statistical analysis and interpretation. The figure summarizes the sequential stages of the research process, including study design, participant recruitment, data collection using a Technology Acceptance Model (TAM)-based questionnaire, variable measurement, data preparation, statistical analysis, covariance-based structural equation modeling (CB-SEM), model validation, and interpretation of findings related to digital transformation readiness among faculty members and students at KSAU-HS.

**Figure 2:**
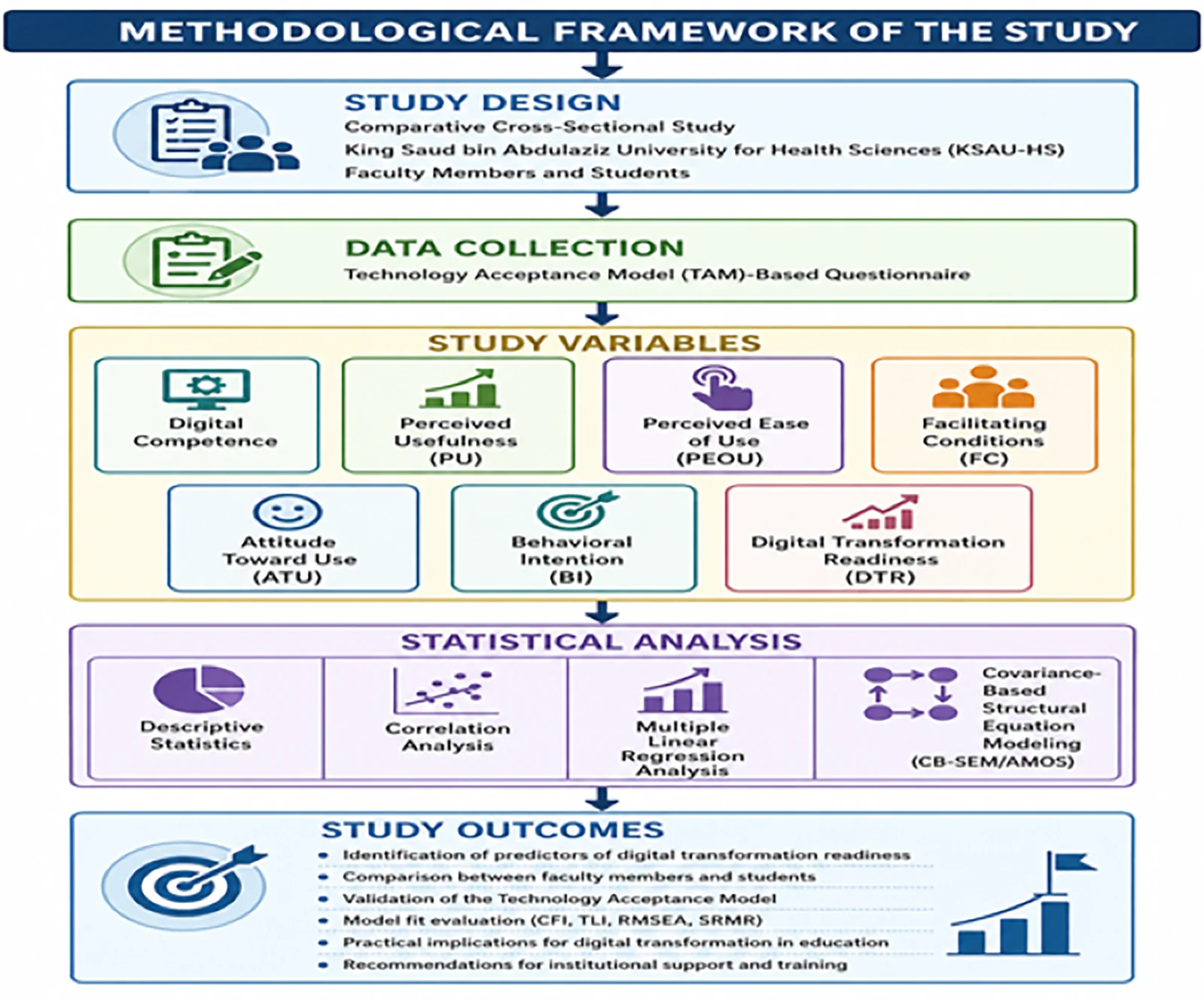
Methodological framework of the study. The framework illustrates the methodological process used to assess digital transformation readiness among faculty members and students at King Saud bin Abdulaziz University for Health Sciences (KSAU-HS), including the study design, data collection procedures, study variables, statistical analyses, and study outcomes.

### Study design

This study used a comparative cross-sectional design among faculty members and students at KSAU-HS to assess and compare their knowledge, attitudes, and readiness for digital transformation. This design enabled efficient data collection from a diverse sample and provided a snapshot of participants’ perspectives at a single point in time, allowing comparisons between the two groups (26, 27).

A conceptual framework was developed based on the (TAM)and t (UTA) and (UT) (23, 24). The framework proposed that knowledge influences attitudes toward digital transformation, which in turn affects readiness, while knowledge also exerts a direct effect on readiness. Structural equation modeling (SEM) and multi-group analysis were used to examine these relationships and compare the proposed model across faculty and student groups. Top of Form.

### Study area/setting

The study was conducted at (KSAU-HS), a medical university with several campuses across Saudi Arabia. The institution includes four schools: Medicine, Nursing, Applied Medical Sciences, and Health Professions. These campuses support a diverse academic environment and are equipped with digital infrastructure aligned with Vision 2030’s goals for integrating digital technologies into education and healthcare. The university’s focus on innovation and modernization made it an appropriate setting for examining digital transformation practices, perceptions, and effects within its academic community.

### Study subjects

The study included students and faculty from all four schools. This diverse group provided perspectives on digital transformation from varied educational and professional backgrounds within healthcare.

### Inclusion criteria

#### Students

Undergraduate and postgraduate learners from the four schools (Medicine, Nursing, Applied Medical Sciences, Health Professions) were included. Their academic and experiential levels help clarify how digital transformation tools are used and accessed in healthcare education.

#### Faculty members

Professors, lecturers, and instructors play a vital role in adapting to digital changes in teaching. Participants represented a cross-section of the KSAU-HS community, providing a holistic view of the effects, challenges, and successes of digital transformation at this healthcare-focused academic institution.

#### Sample size

The Roe Soft online program was used to calculate the sample size, targeting 377 students from an estimated population of 20,000 and 169 faculty members from approximately 300 faculty members across three university cities. This calculation used a 95% confidence level (Z-score of 1.96) and a 5% margin of error, balancing accuracy and feasibility. Anticipating a 50% response rate, oversampling was necessary to achieve the desired sample size.

Sample Size Formula:

$$n=\frac{Z2.p.(1-p)}{e2}$$

N is the required sample size,

Z is the Z-score (1.96 for 95% confidence),

*P* is the estimated proportion of the population (if unknown, use 0.5 for maximum variability),

e is the margin of error (e.g., 0.05 for ±5%).

#### Sampling technique

The study used stratified random sampling and convenience sampling to collect data from the university community. Stratified random sampling involved dividing the population into groups, such as students and faculty, based on university records. Students were then grouped by college—Medicine, Nursing, Applied Medical Sciences, and Health Professions—to reflect their different experiences with digital transformation.

Each group’s sample size was determined by its share of the total population. For instance, if students made up 70% of the university, they also made up 70% of the sample. Each college was included in proportion to its size. Within each group, simple random sampling ensured everyone had an equal chance of being chosen. Combining stratified random sampling with convenience sampling helped ensure the sample represented the university community and made the findings more generalizable.

#### Data collection methods, instruments used, and measurements: the study’s objectives were met using three main tools

Demographic information was collected, including age, gender, education level, marital status, number of children, area of residence, KSAU college attended, and whether they participated in digital transformation workshops.

The Digital Transformation Readiness Scale for Education and Universities measured readiness in infrastructure, resources, adaptability, perceived benefits, digital skills, and intent to use digital tools. Items adapted from Aroean et al. (28) focused on preparedness in education, digital skills, and institutional support, and the scale also included elements from Beetham and Sharpe’s (2013) Digital Capability Framework (29).

#### Psychometric properties

The constructs targeted Cronbach’s alpha values of 0.70 or higher, whereas similar studies often reported values above 0.80. Validity was assessed through expert review, factor analysis, and examination of the relationship between readiness scores and technology use. This approach ensured a thorough and reliable assessment.

The Technology Acceptance Model (TAM) Scale is a widely used tool that focuses on two main ideas: perceived usefulness and perceived ease of use. It helps measure how people accept and use technology. Developed by Davis (25), TAM explains why people adopt and use technology. The updated version also includes Attitude Toward Use and Behavioral Intention to Use. Sample items are: “Digital tools are advantageous for my academic work” (Perceived Usefulness), “My outlook on using digital tools in education is positive” (Attitude Toward Use), “I intend to increase my use of digital tools in educational activities” (Behavioral Intention to Use), and “I find it straightforward to learn new technologies” (Perceived Ease of Use). The original TAM constructs have shown strong internal consistency, with Cronbach’s alpha values typically ranging from 0.70 to 0.95 (23). Later studies, such as Venkatesh and Bala’s (30), have confirmed the model’s reliability and validity across different settings. The adapted TAM Scale is effective for studying technology acceptance in education and for evaluating digital transformation efforts.

The TAM Scale used a 5-point Likert scale from 1 (Strongly Disagree) to 5 (Strongly Agree). For sections with four items, scores were summed, yielding a total of 4 (lowest acceptance) to 20 (highest acceptance). Scores of 16–20 indicated positive attitudes and a strong intention to use technology. Scores of 11–15 indicated neutral attitudes, with some willingness but also some doubts. Scores of 4–10 indicated negative attitudes and concerns about technology’s usefulness or ease of use.

The Teachers’ Digital Competence (TDC) Scale, developed by the European Commission’s Joint Research Center as part of the Dig Comp Edu framework, measures teachers’ digital skills and how well they use digital tools in teaching. It assesses skills such as creating digital content, digital communication, using digital teaching strategies, understanding data, keeping online activities safe, conducting assessments, and improving digital skills through professional development.

The TDC Scale has 21 items across seven areas. It measures how ready and able teachers are to use digital resources in education. Each item is rated from 1 (“Not Competent”) to 5 (“Highly Competent”), giving a detailed view of teachers’ digital skills. The TDC Scale has demonstrated robust reliability, with Cronbach’s alpha of 0.80 or higher, indicating strong internal consistency across domains. It also demonstrates construct and content validity, corresponding with established digital competence frameworks, making it a dependable tool for evaluating educators’ digital readiness.

The Students' Digital Competence (SDC) Scale was adapted from DigComp 2.2: The Digital Competence Framework for Citizens, as described by Vuorikari et al. (31). It measures students’ digital skills across education levels, including how they use digital environments, manage digital tools, and follow safe and ethical online practices. It consists of five key areas, each comprising five items, focusing on fundamental digital skills, information literacy, communication, problem-solving, and ethical awareness. It uses a 5-point Likert scale, ranging from 1 (“Not Competent”) to 5 (“Highly Competent”), to measure students’ self-assessment. This scale had a Cronbach’s alpha of 0.85 or higher, indicating good internal consistency. It aligns with major digital competence frameworks and effectively measures how well students adapt and perform in digital environments.

#### Reliability for the current study

Cronbach’s alpha was 0.903 for Students’ Digital Competence, 0.896 for Students’ TAM, 0.901 for Students’ Digital Transformation Readiness, and 0.892 for Faculty’s Digital Competence.

### Data collection procedure

The data collection approach for the KSAU-HS digital transformation study was systematic and multifaceted. It aimed to secure reliable responses from students and faculty in all four academic colleges. After obtaining approvals from the research unit, King Abdullah International Medical center (KAIMRC), and the (International review Board) IRB, the team created a structured English-language survey in Google Forms. The survey asked about the benefits, challenges, and effects of digital transformation on academic and administrative work. A pilot study tested the survey for clarity, relevance, and reliability.

Once the survey was ready, electronic invitations were sent to students and faculty through official university emails and digital platforms, including the online learning management system. This approach made sure everyone across the campuses had access and encouraged participation. The survey was open for four months, giving everyone enough time to complete it. Weekly reminders were sent to encourage participation and improve response rates.

Participation was tracked during data collection. If response rates were low, follow-up reminders were sent to non-responders, and additional outreach, such as college announcements, was used to increase engagement.

All responses were kept anonymous to protect confidentiality. The data was stored securely in a password-protected database accessible only by the research team.

Data Verification: After the survey closed, the team carefully checked the data for completeness. Any invalid or incomplete responses were removed before analysis. This step helped ensure the final dataset was accurate and representative of the university community.

This organized approach to data collection aimed to create a complete dataset that reflects the different views on digital transformation among students and faculty at KSAU-HS.

### Data management and analysis plan

The data analysis was conducted using the latest version of Statistical Package for the Social Sciences. (SPSS). First, descriptive statistics summarized demographic details and responses about knowledge, attitudes, and readiness among faculty and students, using means, standard deviations, frequencies, and percentages. To compare the two groups, independent-samples t-tests were used, or Mann–Whitney U tests if the data were not normally distributed. To examine relationships within each group, Pearson’s correlation or Spearman’s rho (for nonnormal data) was used to measure the strength and direction of associations. Multiple regression analysis examined how well knowledge and attitudes predicted readiness for digital transformation, while controlling for demographic factors. Chi-square tests were also used to determine whether demographic characteristics were associated with readiness or attitudes. This approach provided a thorough overview of the main variables and helped make recommendations to improve digital transformation readiness.

### Statistical analysis

All statistical analyses were conducted using SPSS for Windows version 20.0 (SPSS, Chicago, IL). Continuous data were normally distributed and presented as mean ± standard deviation (SD), while categorical data were presented as numbers and percentages. The chi-square test, or Fisher’s exact test when needed, compared categorical variables. The correlation coefficient test assessed the relationship between two continuous variables, and linear regression was used to predict one variable from the other. The internal consistency (reliability) of the questionnaires was also calculated, and statistical significance was set at *p* < 0.05.All statistical results reported in the manuscript were verified against the final analytical outputs to ensure consistency in coefficients, significance levels, confidence intervals, and effect size estimates across all tables, figures, and narrative descriptions.

## Results

Table 1 shows that faculty members were predominantly middle-aged. The highest proportions were in the 50–59-year (30.3%) and 40–49-year (28.0%) age groups. The mean age was 47.3 ± 10.3 years. Most faculty had 10–20 years of experience (38.9%) or more than 20 years (37.7%). This indicates a generally well-experienced sample (mean = 16.7 ± 8.3 years).

**Table 1:** Distribution of the age and experience years among faculty (*N* = 350), and students (*N* = 377).

Participant characteristic/category	n	%
Faculty members’ Age (Years)		
<30	4	1.1
30–39	92	26.3
40–49	98	28.0
50–59	106	30.3
60 or more	50	14.3
Mean ± SD	47.3 ± 10.3	
Faculty Members’ Experience (Years)		
<10	82	23.4
10–20	136	38.9
>20	132	37.7
Mean ± SD	16.7 ± 8.3	
Students’ Age (Years)		
20 or lower	97	25.7
>20	280	74.3
Mean ± SD	21.5 ± 1.7	
Students’ Experience (Years)		
<2	110	29.2
2–4	175	46.4
>4	92	24.4
Mean ± SD	2.9 ± 1.9	

Similarly, among students, the majority were older than 20 years (74.3%), with a mean age of 21.5 ± 1.7 years. Regarding experience, nearly half (46.4%) had 2–4 years, while smaller proportions had less than 2 years (29.2%) or more than 4 years (24.4%), with an average of 2.9 ± 1.9 years. Thus, the table shows a mature, experienced faculty group alongside relatively young students with moderate experience.

 Table 2 demonstrates statistically significant differences between faculty members and students across all examined demographic characteristics (*p* < 0.001). Females constituted the majority in both groups, but the proportion was higher among students (84.9%) than among faculty members (57.4%). Faculty members were predominantly highly qualified, with most holding Master’s, Doctorate, or PhD degrees, whereas students were more concentrated at the bachelor’s level or in earlier stages of postgraduate education.

**Table 2:** Comparison between the demographic characteristics of the faculty (*N* = 350) and students (*N* = 377).

Demographic characteristic/category	Faculty members		Students		Chi – square/Fisher’s exact test	
	n	%	n	%	X^2^	P
Gender						
Male	149	42.6	57	15.1		
Female	201	57.4	320	84.9	67.358	<0.001***
Educational Level						
Bachelor’s	0	0.0	92	24.4		
Master’s	126	36.0	100	26.5		
Doctorate	131	37.4	98	26.0		
PhD	93	26.6	87	23.1	99.081	<0.001***
Marital Status						
Married	133	38.0	66	17.5		
Single	119	34.0	306	81.2		
Divorced/Widowed	98	28.0	5	1.3	188.065	<0.001***
Area of Residence						
Central Region	122	34.9	54	14.3		
Eastern Region	110	31.4	37	9.8		
Western Region	118	33.7	286	75.9	131.565	<0.001***
KSAU College Affiliation						
Applied Medical Sciences	50	14.3	19	5.0		
Dentistry	45	12.9	23	6.1		
Health Profession	86	24.6	16	4.2		
Medicine	45	12.9	31	8.2		
Nursing	64	18.3	267	70.8		
Pharmacy	60	17.1	13	3.4		
Science and Health Professions	0	0.0	8	2.1	233.742	<0.001***

***Statistically significant at *p* < 0.001.

Marital status also differed: most students were single (81.2%), whereas faculty members were more evenly distributed and more often married or previously married. Most students resided in the Western Region (75.9%), whereas faculty members were more evenly spread across the Central, Eastern, and Western regions. In terms of college affiliation, students were mainly from the Nursing college (70.8%), while faculty members were distributed across Health Professions, Nursing, and Pharmacy.

 Table 3 shows clear differences between faculty and students in their use and views of digital tools (*p* < 0.001), except for device types, where both groups were similar. More students (61.8%) used digital tools daily than faculty (44.6%), while faculty had more weekly and monthly users.

**Table 3:** Comparison between the device usage among faculty (350) and students (377).

Demographic characteristic/category	Faculty members		Students		Chi – square/Fisher’s exact test	
	n	%	n	%	X^2^	P
How often do you use digital tools for your academic or professional activities?						
Daily	156	44.6	233	61.8		
Weekly	109	31.1	77	20.4		
Monthly	85	24.3	48	12.7		
Rarely	0	0.0	17	4.5		
Never	0	0.0	2	0.5	49.105	<0.001***
How would you rate your own digital competence?						
Very low	0	0.0	8	2.1		
Low	0	0.0	18	4.8		
Moderate	115	32.9	149	39.5		
High	119	34.0	119	31.6		
Very high	116	33.1	83	22.0	34.897	<0.001***
Devices you use						
PC/Laptop	268	76.6	285	75.6	0.095	0.758
Tablet	298	85.1	309	82.0	1.332	0.248
Smartphone	290	82.9	302	80.1	0.908	0.341
Internet Reliability						
Very Unreliable	2	0.6	8	2.1		
Somewhat Unreliable	2	0.6	7	1.9		
Neutral	119	34.0	73	19.4		
Somewhat Reliable	100	28.6	93	24.7		
Very Reliable	127	36.3	196	52.0	31.433	<0.001***
Motivation Level						
Not at All	1	0.3	26	6.9		
Slightly Motivated	1	0.3	30	8.0		
Moderately Motivated	112	32.0	115	30.5		
Motivated	102	29.1	98	26.0		
Very Motivated	134	38.3	108	28.6	52.260	<0.001***
Formal DT Training						
No	160	45.7	296	78.5		
Yes	190	54.3	81	21.5	83.515	<0.001***
Confidence in Digital Adaptation						
Not at All Confident	0	0.0	16	4.2		
Slightly Confident	111	31.7	83	22.0		
Moderately Confident	125	35.7	132	35.0		
Very Confident	107	30.6	110	29.2		
Extremely Confident	7	2.0	36	9.5	38.882	<0.001***

***Statistically significant at *p* < 0.001.

Faculty rated their digital skills higher, with more in the high or very high categories. Most students rated themselves as moderate, and some reported low competence. Both groups used similar devices, suggesting similar access to technology.

More students (52.0%) than faculty (36.3%) felt their internet was very reliable. Faculty were generally more motivated, with more in the ‘very motivated’ group, while students were more likely to report lower motivation.

There was a clear difference in digital transformation training: 54.3% of faculty received training, compared with only 21.5% of students. Both groups were generally confident about adapting to digital tools, but students’ confidence varied more, with some feeling less confident and others extremely confident.

In summary, students use digital tools more often and view the internet as more reliable, while faculty report higher digital skills, motivation, and more formal training in digital technology.

 Table 4 shows the distribution of digital competence levels among students and faculty across different domains. Most students had high digital competence, especially in *Communication & Collaboration* (54.9%), *Safety & Digital Citizenship* (53.6%), and *Device & Software Operations* (53.1%). Only about 10%–12% of students had low competence in any domain. In total, over half of the students (50.4%) had high digital competence, while 39.8% were at a moderate level.

**Table 4:** Distribution of the digital competence domains and overall level.

Digital competence domain/overall level	Low		Moderate		High	
	n	%	n	%	n	%
Students’ Domains						
Device & Software Operations	40	10.6	137	36.3	200	53.1
Information & Data Literacy	40	10.6	149	39.5	188	49.9
Communication & Collaboration	38	10.1	132	35.0	207	54.9
Problem-Solving & Adaptability	42	11.1	139	36.9	196	52.0
Safety & Digital Citizenship	45	11.9	130	34.5	202	53.6
Overall, the students’ level	37	9.8	150	39.8	190	50.4
Faculty Members’ Domains						
Content Creation & Pedagogy	8	2.3	216	61.7	126	36.0
Communication & Collaboration	25	7.1	141	40.3	184	52.6
Information & Data Literacy	25	7.1	144	41.1	181	51.7
Safety & Digital Citizenship	30	8.6	154	44.0	166	47.4
Assessment & Feedback	24	6.9	153	43.7	173	49.4
Professional Development	21	6.0	145	41.4	184	52.6
Overall, the faculty members’ level	5	1.4	248	70.9	97	27.7

Faculty members showed a different trend, with most falling into the moderate competence category. The highest rates of high competence were in *Communication & Collaboration* (52.6%), *Professional Development* (52.6%), and *Information & Data Literacy* (51.7%). In Content Creation & Pedagogy, most faculty (61.7%) were at a moderate level. Overall, 70.9% of faculty had moderate digital competence, 27.7% had high competence, and only 1.4% had low competence.

Overall, these findings suggest that students tend to exhibit higher levels of digital competence across domains. In summary, students generally demonstrate higher digital competence across domains than faculty members do. Faculty competence is mostly moderate, particularly in teaching and content-related areas.

 Table 5 highlights clear, statistically significant differences between faculty and students across all areas of the (TAM) and Digital Transformation Readiness (DTR) (*p* < 0.001). Faculty consistently reported higher proportions in the high-level category, especially in *Self-Efficacy* (86.3%), *Perceived Benefits* (86.9%), and *Infrastructure Readiness* (85.7%), indicating stronger technology acceptance and readiness than students.

**Table 5:** Comparison of technology acceptance model (TAM) and digital transformation readiness (DTR) domains between faculty members and students with effect size.

Domains	Faculty Members Low *n* (%)	Moderate *n* (%)	High *n* (%)	Students Low *n* (%)	Moderate *n* (%)	High *n* (%)	X²	*P*-value	Cramér’s V
Technology Acceptance Model (TAM)									
Perceived Usefulness	2 (0.6)	60 (17.1)	288 (82.3)	25 (6.6)	88 (23.3)	264 (70.0)	24.965	<0.001	0.131
Perceived Ease of Use	3 (0.9)	67 (19.1)	280 (80.0)	28 (7.4)	102 (27.1)	247 (65.5)	28.513	<0.001	0.140
Attitude Toward Use	2 (0.6)	68 (19.4)	280 (80.0)	26 (6.9)	97 (25.7)	254 (67.4)	25.967	<0.001	0.134
Behavioral Intention to Use	3 (0.9)	66 (18.9)	281 (80.3)	27 (7.2)	89 (23.6)	261 (69.2)	22.379	<0.001	0.124
Facilitating Conditions	6 (1.7)	58 (16.6)	286 (81.7)	34 (9.0)	99 (26.3)	244 (64.7)	32.678	<0.001	0.150
Digital Transformation Readiness (DTR)									
Self-Efficacy	2 (0.6)	46 (13.1)	302 (86.3)	30 (8.0)	71 (18.8)	276 (73.2)	30.050	<0.001	0.144
Perceived Benefits	2 (0.6)	44 (12.6)	304 (86.9)	27 (7.2)	69 (18.3)	281 (74.5)	27.021	<0.001	0.136
Openness to Change	2 (0.6)	48 (13.7)	300 (85.7)	31 (8.2)	72 (19.1)	274 (72.7)	30.502	<0.001	0.145
Behavioral Intention	3 (0.9)	52 (14.9)	295 (84.3)	29 (7.7)	82 (21.8)	266 (70.6)	28.377	<0.001	0.140
Institutional Support	6 (1.7)	54 (15.4)	290 (82.9)	29 (7.7)	81 (21.5)	267 (70.8)	20.490	<0.001	0.119
Infrastructure Readiness	5 (1.4)	45 (12.9)	300 (85.7)	31 (8.2)	76 (20.2)	270 (71.6)	27.334	<0.001	0.137
Overall Level	2 (0.6)	48 (13.7)	300 (85.7)	27 (7.2)	75 (19.9)	275 (72.9)	60.568	<0.001***	0.204

***Statistically significant at *p* < 0.001.

Students reported fewer high-category responses and more moderate- or low-category responses across all domains, indicating lower technology acceptance and digital readiness than faculty.

While all differences are statistically significant, most effect sizes (Cramér’s V) are small. Only overall readiness and acceptance show a near-moderate effect (V = 0.204), suggesting the practical gap is limited and mainly concentrated in the aggregated scores.

Faculty demonstrate greater technology acceptance and digital readiness than students, but these differences, while real, suggest a limited practical impact.

 Figure 3 shows how students’ digital skills are distributed across six main areas, with most students achieving a high level in each area. About 49.9% to 54.9% of students are in the “high” category, indicating strong overall digital abilities. Communication and collaboration have the highest score (54.9%), followed by safety and digital citizenship (53.6%) and device and software operations (53.1%). This suggests that students are especially skilled at practical, interactive digital tasks.

**Figure 3:**
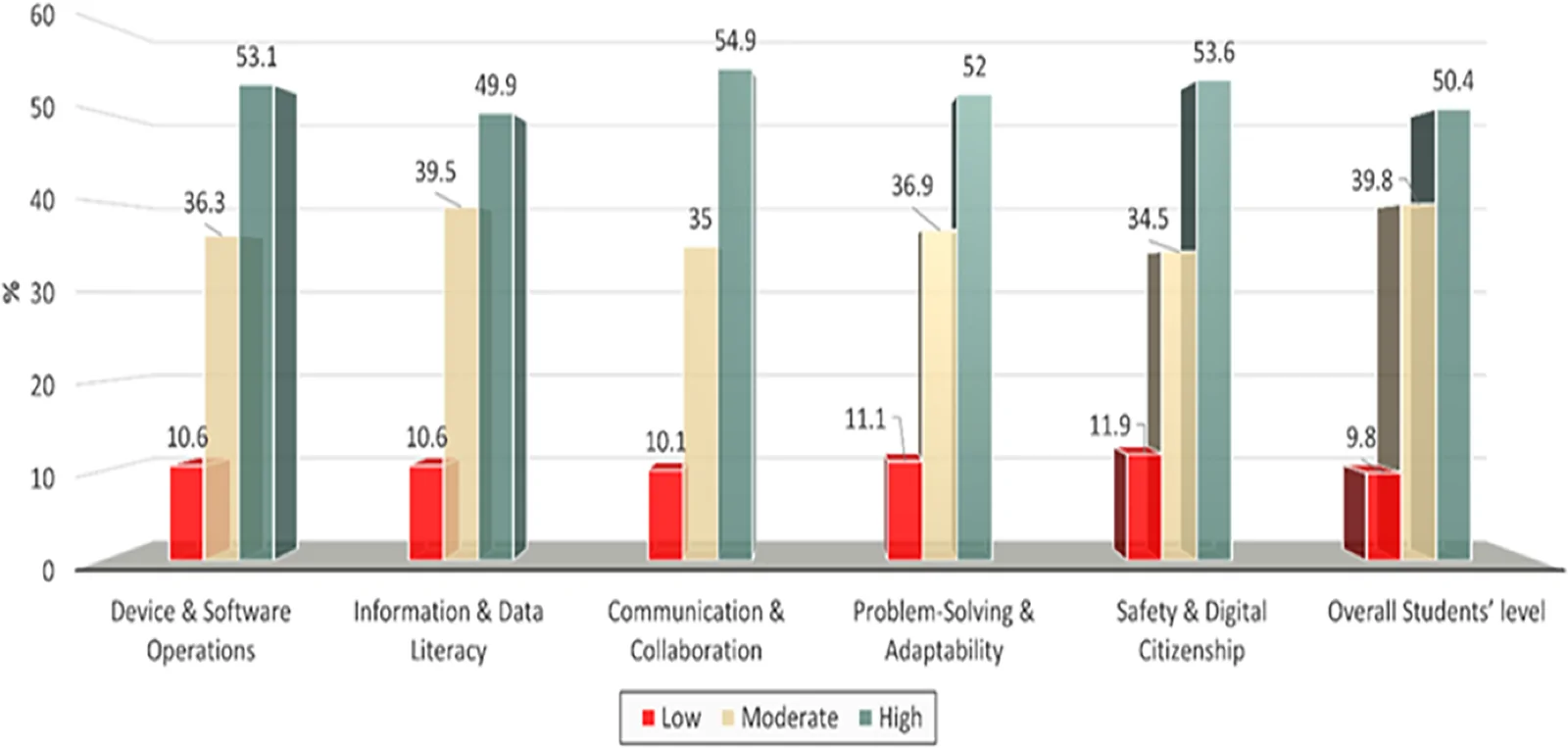
The distribution of students’ digital competency levels.

Many students fall into the moderate-competency group, with percentages ranging from 34.5% to 39.8% across all areas. This means a significant number have basic, not advanced, skills. More students are at the moderate level in information and data literacy (39.5%) and overall competency (39.8%). These results point to possible gaps in students’ analytical and information-processing skills, which may limit academic work and evidence-based practice.

The percentage of students classified as having low competency is consistently low, at 9%. Only a small percentage of students have low digital skills, ranging from 9.8% to 11.9%, indicating that most have a solid foundation. Slightly more students have low scores in safety and digital citizenship (11.9%) and in problem-solving and adaptability (11.1%). This suggests that a few students may need extra help with critical thinking, digital responsibility, and problem-solving. Overall, students’ digital competence is strong, but targeted educational interventions may enhance higher-order skills, particularly in information literacy and problem-solving.

Figure 4 shows faculty members’ digital competency levels (low, moderate, and high) across six key domains. The pattern is more varied than among students, with the moderate level dominating several domains. This is especially evident in content creation and pedagogy, where moderate competency reaches 61.7%, while the high level is lower at 36%. These results suggest that faculty members have adequate foundational skills in instructional design and digital pedagogy, but many have not yet reached advanced proficiency.

**Figure 4:**
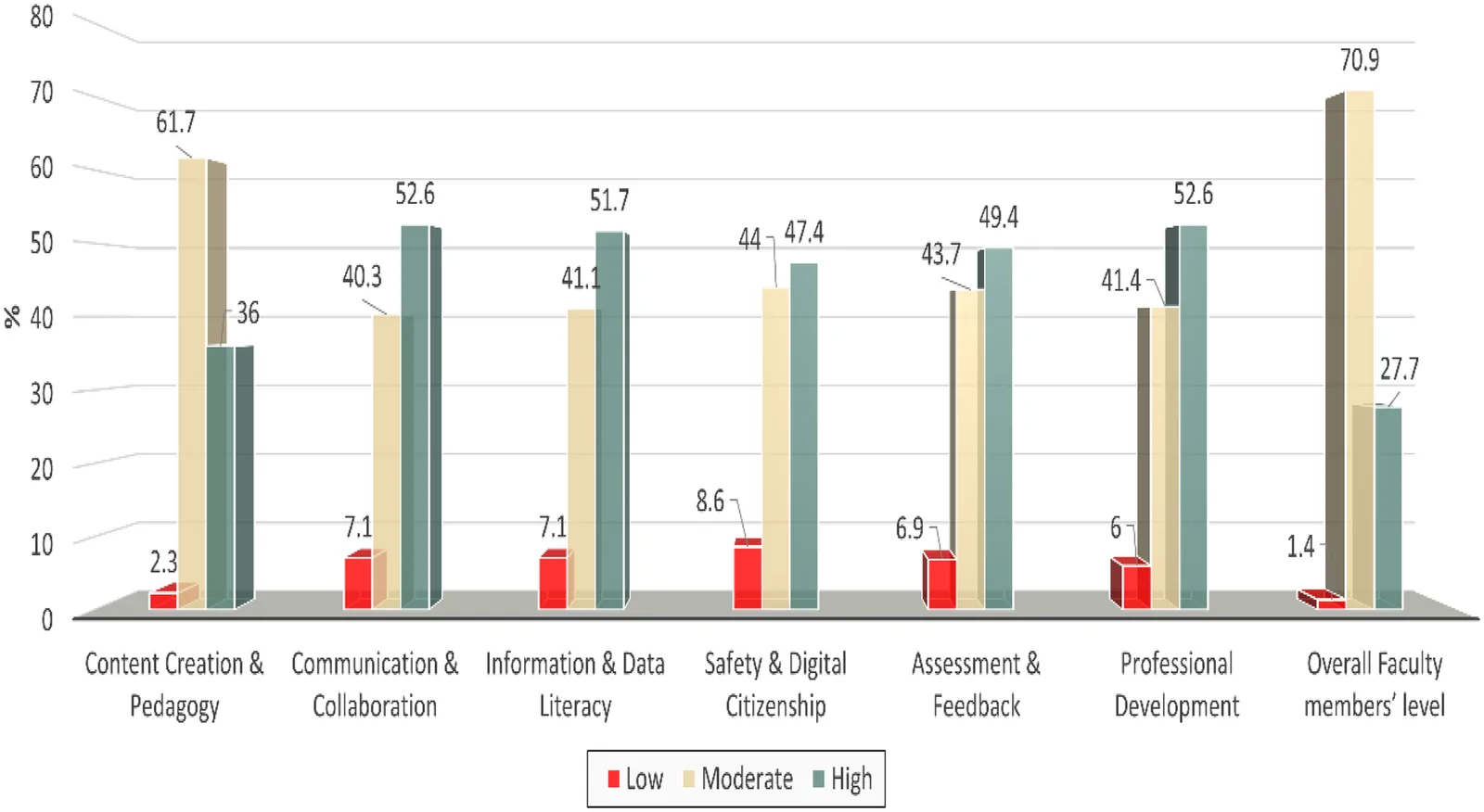
Distribution of the faculty members’ digital competence domains and overall level.

In the remaining domains, higher competency levels are more common. The proportion of faculty classified as “high” ranges from 47.4% to 52.6%, including communication and collaboration (52.6%), professional development (52.6%), information and data literacy (51.7%), assessment and feedback (49.4%), and safety and digital citizenship (47.4%). These results reflect strong capabilities in communication, continuous learning, information handling, evaluation, and ethical practice.

The percentage of faculty members with low competency remains low across all domains (1.4%–8.6%), indicating overall digital readiness. Slightly higher low-level percentages are observed in safety and digital citizenship (8.6%) and information and data literacy (7.1%), suggesting that a minority of faculty may still need support with digital responsibility and data skills.

Overall, faculty members are concentrated in the moderate category (70.9%), followed by the high category (27.7%) and the low category (1.4%). This distribution indicates that faculty members generally possess adequate digital competence, but their skills remain more developed than advanced. In summary, faculty members possess solid foundational and functional digital skills, particularly in communication and professional development. However, the prevalence of moderate competency, especially in content creation and pedagogy, highlights the need for targeted professional development to strengthen advanced digital teaching competencies and foster greater innovation in educational practice.

 Table 6 shows statistically significant differences between faculty and students across all TAM domains (*p* < 0.001). Faculty were more likely to fall into the high-level category, particularly for *Perceived Usefulness* (82.3%), *Perceived Ease of Use* (80.0%), and *Behavioral Intention to Use* (80.3%), indicating higher acceptance overall.

**Table 6:** Comparison of the technology acceptance model domains.

Technology Acceptance Model domains	Faculty members						Students						Chi – square/Fisher’s exact test	
	Low		Moderate		High		Low		Moderate		High			
	n	%	n	%	n	%	n	%	n	%	n	%	X^2^	P
Domains														
Perceived Usefulness	2	0.6	60	17.1	288	82.3	25	6.6	88	23.3	264	70.0	24.965	<0.001***
Perceived Ease of Use	3	0.9	67	19.1	280	80.0	28	7.4	102	27.1	247	65.5	28.513	<0.001***
Attitude Toward Use	2	0.6	68	19.4	280	80.0	26	6.9	97	25.7	254	67.4	25.967	<0.001***
Behavioral Intention to Use	3	0.9	66	18.9	281	80.3	27	7.2	89	23.6	261	69.2	22.379	<0.001***
Facilitating Conditions	6	1.7	58	16.6	286	81.7	34	9.0	99	26.3	244	64.7	32.678	<0.001***

***Statistically significant at *p* < 0.001.

In contrast, students were less likely to be in the high category and more likely to be in the moderate and low categories, reinforcing the gap noted above. This difference is most noticeable in *Facilitating Conditions*, where 9.0% of students reported low levels, compared to just 1.7% of faculty. Overall, these results suggest that faculty members see digital technologies as more useful, easier to use, and more acceptable than students. The consistent differences point to a gap in technology acceptance that favors faculty.

 Figure 5 compares digital readiness between faculty members and students using the Technology Acceptance Model (TAM) constructs and facilitating conditions. Clear differences appear in their perceived digital competence.

**Figure 5:**
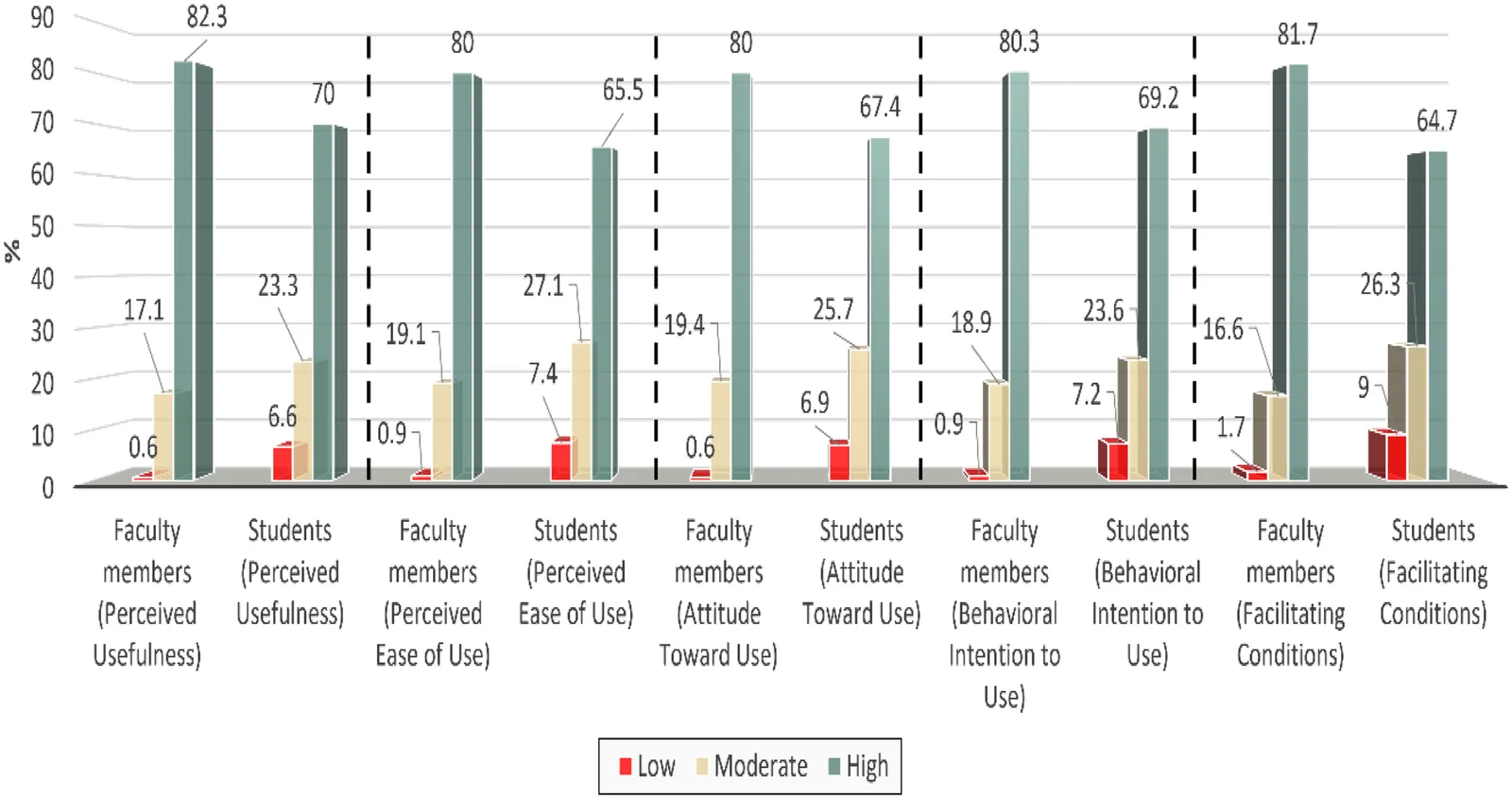
Comparison of the digital transformation readiness domains and overall level.

Faculty members show higher competency across all domains than students do. For example, 82.3% of faculty report high perceived usefulness, while only 70% of students do. A similar trend holds for perceived ease of use, with 80% of faculty in the high category compared with 65.5% of students.

Faculty members also have stronger attitudes toward use: 80% report high levels, compared with 67.4% of students. They report a high behavioral intention to use at 80.3%, while students reach only 69.2%. This suggests faculty are more likely than students to integrate digital technologies.

Faculty report more support and better infrastructure (81.7% high), compared with students (64.7%). This may reflect differences in access to resources, training, or digital confidence.

Students show higher proportions in moderate and low categories across constructs. Among them, perceived usefulness and ease of use range from 23.3% to 27.1%, with faculty showing lower moderate percentages. Low-level responses are more pronounced among students (up to 9%), while among faculty, they remain below 2%.

In summary, the figure shows that faculty are more digitally ready. They have higher perceived usefulness, ease of use, positive attitudes, and intentions. Students are generally competent but show more variability and need support to build digital confidence and engagement. These findings highlight the need for targeted interventions to improve students’ digital readiness and for leveraging faculty expertise to support their development.

 Table 7 shows a significant difference in Digital Transformation Readiness (DTR) between faculty and students across all domains (*p* < 0.001). Faculty more often fell into the high-level category, especially for *Perceived Benefits* (86.9%), *Self-Efficacy* (86.3%), and *Infrastructure Readiness* (85.7%).

**Table 7:** Comparison of the digital transformation readiness domains and overall level.

Digital transformation readiness domain/overall level	Faculty members (*N* = 350)						Students(*N* = 377)						Chi – square/Fisher’s exact test	
	High		Low		Moderate		High		Low		Moderate			
	n	%	n	%	n	%	n	%	n	%	n	%	X^2^	P
Domains														
Self-Efficacy	2	0.6	46	13.1	302	86.3	30	8.0	71	18.8	276	73.2	30.050	<0.001***
Perceived Benefits	2	0.6	44	12.6	304	86.9	27	7.2	69	18.3	281	74.5	27.021	<0.001***
Openness to Change	2	0.6	48	13.7	300	85.7	31	8.2	72	19.1	274	72.7	30.502	<0.001***
Behavioral Intention	3	0.9	52	14.9	295	84.3	29	7.7	82	21.8	266	70.6	28.377	<0.001***
Institutional Support	6	1.7	54	15.4	290	82.9	29	7.7	81	21.5	267	70.8	20.490	<0.001***
Infrastructure Readiness	5	1.4	45	12.9	300	85.7	31	8.2	76	20.2	270	71.6	27.334	<0.001***
Overall level	2	0.6	48	13.7	300	85.7	27	7.2	75	19.9	275	72.9	60.568	<0.001***

***Statistically significant at *p* < 0.001.

Students, on the other hand, had fewer high-level responses and more moderate and low responses across all domains, especially in *Openness to Change* (8.2%) and *Infrastructure Readiness* (8.2%), whereas faculty reported very few low-level responses.

This gap is clear in the overall results: 85.7% of faculty show high readiness, while only 72.9% of students do. More students fall into the moderate and low readiness categories.

To summarize, the findings show that faculty are better prepared for digital transformation across all areas, including confidence, perceived value, and institutional and infrastructural support. These consistent results point to a clear advantage in faculty readiness for digital transformation.

 Table 8 shows that several socio-demographic and digital-related factors are linked to the main study constructs (SDC, PU, PEOU, ATU, BI, FC, and DTR). Motivation level was the strongest and most consistent predictor, with highly significant relationships across all areas (*p* < 0.001). This highlights its key role in digital transformation readiness and technology acceptance. Digital competence was also important, showing significant links with perceived usefulness (PU; *p* = 0.028), perceived ease of use (PEOU; *p* < 0.001), attitude toward use (ATU; *p* = 0.003), behavioral intention (BI; *p* < 0.001), and digital transformation readiness (DTR; *p* < 0.001). Confidence was strongly associated with most constructs, including SDC, PU, PEOU, ATU, and BI (all *p* < 0.001), suggesting that psychological readiness plays a significant role in technology adoption.

**Table 8:** Summary of significant associations between students’ characteristics and study variables.

Variable	SDC	PU	PEOU	ATU	BI	FC	DTR
Age	0.238	0.726	0.822	0.989	0.910	0.478	0.824
Gender	0.352	**0**.033*	0.088	**0**.011*	0.307	**0**.003*	0.402
Educational Level	0.985	0.814	0.672	0.905	0.910	0.978	0.627
Marital Status	**<0**.001***	0.222	0.218	0.199	0.156	0.100	**0**.003*
Area of Residence	**<0**.001***	0.096	0.250	0.364	**0**.004*	**0**.019*	0.404
College Affiliation	0.452	0.142	0.479	0.106	0.053	0.341	0.239
Academic Experience	0.173	0.268	**<0**.001***	0.059	0.235	0.174	0.079
Digital Tool Use	0.431	0.193	0.240	0.155	0.064	0.170	0.296
Digital Competence	0.208	**0**.028*	**<0**.001***	**0**.003*	**<0**.001***	0.128	**<0**.001***
Uses PC/Laptop	0.832	0.327	0.187	0.053	0.488	0.051	0.311
Uses Tablet	0.194	0.142	0.299	0.082	0.243	**<0**.001***	**0**.002*
Uses Smartphone	0.417	0.373	0.739	0.428	0.358	0.908	0.307
Internet Reliability	0.443	**0**.042*	0.302	0.432	0.072	0.129	0.417
Motivation Level	**<0**.001***	**<0**.001***	**<0**.001***	**<0**.001***	**<0**.001***	**<0**.001***	**<0**.001***
Formal DT Training	0.362	0.282	0.080	0.170	0.172	0.168	0.297
Confidence	**<0**.001***	**0**.003*	**<0**.001***	**<0**.001***	**<0**.001***	0.052	0.439

Abbreviations: SDC, student digital competence; PU, perceived usefulness; PEOU, perceived ease of use; ATU, attitude toward use; BI, behavioral intention; FC, facilitating conditions; DTR, digital transformation readiness; DT, digital transformation.

Note: Values presented are *p*-values. Bold values indicate statistically significant associations.

* *p* < 0.05.

*** *p* < 0.001.

Gender differences appeared in PU (*p* = 0.033), ATU (*p* = 0.011), and facilitating conditions (FC; *p* = 0.003). Marital status was linked to SDC (*p* < 0.001) and DTR (*p* = 0.003). Where people lived was associated with SDC (*p* < 0.001), BI (*p* = 0.004), and FC (*p* = 0.019), indicating that context matters. Academic experience was only associated with PEOU (*p* < 0.001), suggesting that greater experience can make digital systems feel easier to use. For technology access, tablet use was linked to FC (*p* < 0.001) and DTR (*p* = 0.002), and reliable internet was connected to PU (*p* = 0.042). Educational level, college affiliation, smartphone use, and formal digital transformation training were not significantly associated with most constructs. Overall, these results show that motivation, cognitive factors, and competence are more important than demographic or structural factors for digital transformation readiness and technology acceptance.

 Figure 6 shows a heatmap of statistically significant links between independent variables (rows) and key study constructs (columns). The constructs are self-digital competence (SDC), perceived usefulness (PU), perceived ease of use (PEOU), attitude toward use (ATU), behavioral intention (BI), facilitating conditions (FC), and digital transformation readiness (DTR). Darker colors indicate higher statistical significance (*p < 0.05; **p *<* 0*.001*). Motivation shows the strongest association across all constructs. Digital competence and confidence also exhibit several significant links, particularly with the main variables in the Technology Acceptance Model (TAM). Demographic and structural variables show only limited, specific effects.

**Figure 6:**
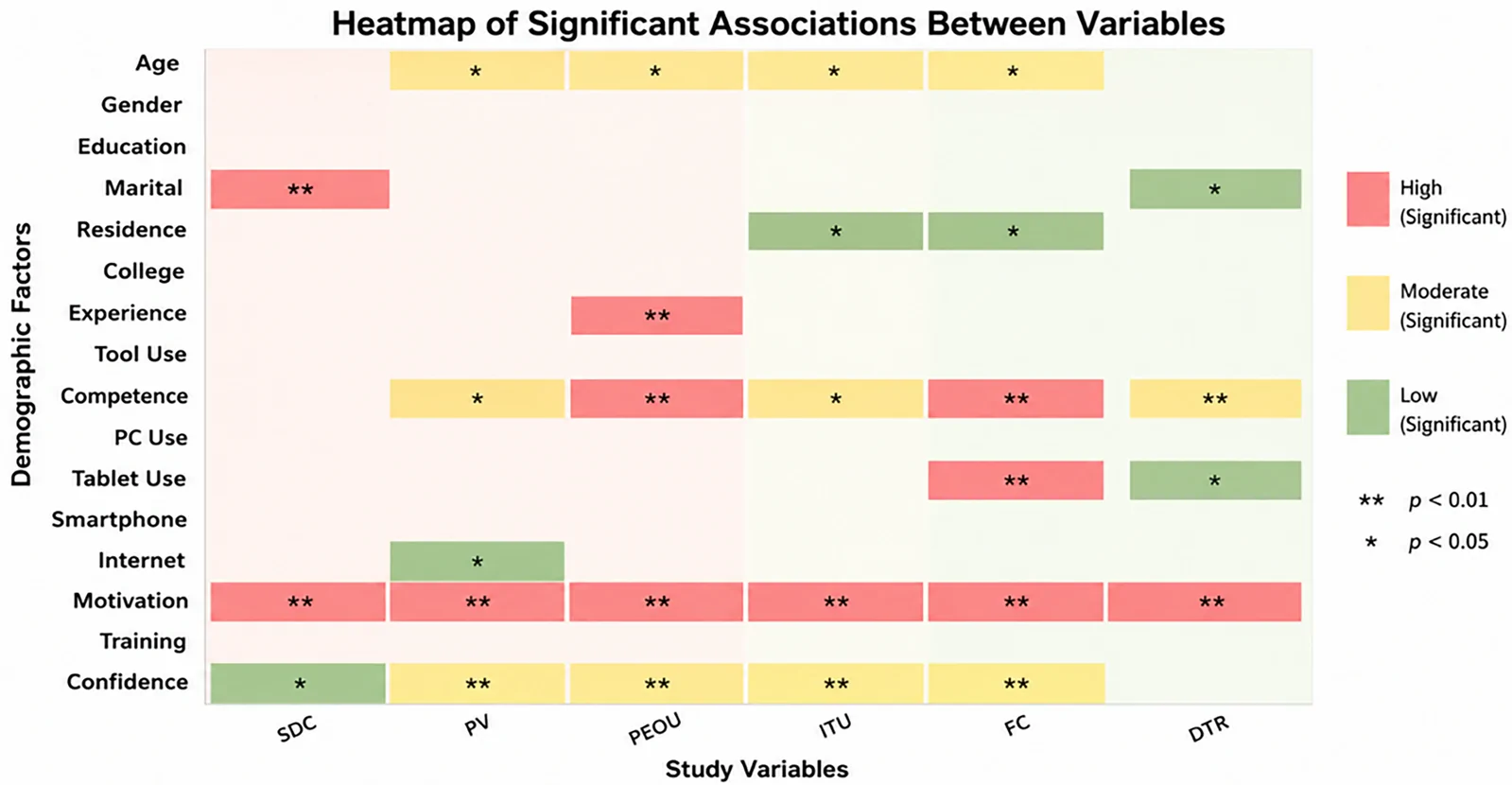
Heatmap of significant associations between students’ demographic, technological, and psychological factors and digital transformation constructs. Cells marked with * indicate *p* < 0.05, and ** indicate *p* < 0.001.

 Table 9 shows that chi-square analysis identified several significant associations between socio-demographic factors and areas of digital transformation. Confidence in digital adaptation and motivation were the most consistent predictors, with strong associations across SDC level, perceived usefulness, perceived ease of use, attitude toward use, behavioral intention, facilitating conditions, and digital transformation readiness (DTR level) in multiple analyses. Age was also often significant across SDC level, perceived ease of use, attitude toward use, behavioral intention, and facilitating conditions. Formal training was important across several areas, including perceived usefulness, attitude toward use, facilitating conditions, and DTR level. Other factors, including college affiliation, marital status, PC or laptop use, area of residence, gender, educational level, and frequency of digital tool use, were significant in specific domains. For example, college affiliation was linked to SDC level and perceived usefulness; marital status to perceived usefulness and attitude toward use; PC or laptop use to perceived ease of use; area of residence to perceived ease of use and facilitating conditions; gender to perceived ease of use and DTR level; educational level to SDC level; and frequency of digital tool use to behavioral intention. These results show which predictors are shared and how important they are across different digital transformation domains.

**Table 9:** Significant associations between socio-demographic variables and digital transformation domains.

Outcome	Significant Predictors	*χ*²	*P*-value
SDC Level	Age	23.769	0.003*
	Educational Level	9.969	0.041*
	College Affiliation	15.627	0.015*
	Confidence in Digital Adaptation	14.143	0.028*
Perceived Usefulness	Marital Status	7.212	0.030*
	College Affiliation	13.409	0.039*
	Formal DT Training	3.006	0.038*
	Confidence in Digital Adaptation	30.899	<0.001**
Ease of Use	Age	30.210	<0.001**
	Gender	3.227	0.025*
	Area of Residence	5.112	0.013*
	Uses PC/Laptop	5.291	0.002**
	Confidence in Digital Adaptation	18.777	0.005**
Attitude Toward Use	Age	9.772	0.014
	Marital Status	6.811	0.006**
	Formal Digital Training	2.445	0.019*
	Confidence in Digital Adaptation	27.435	<0.001**
Behavioral Intention	Age	30.716	<0.001**
	Digital Tool Use Frequency	8.597	0.006**
	Motivation Level	120.243	<0.001**
	Confidence in Digital Adaptation	20.186	0.003**
Facilitating Conditions	Age	16.639	0.034*
	Area of Residence	8.954	0.008**
	Motivation Level	61.497	<0.001**
	Formal digital Training	8.019	0.018*
DTR Level	Gender	3.247	0.006**
	Motivation Level	30.886	<0.001**
	Formal DT Training	5.847	0.037*
	Confidence in Digital Adaptation	26.107	<0.001**

*Statistically significant at *p* < 0.05.

**Statistically significant at *p* < 0.01.

 Figure 7 shows heatmaps revealing clearer differences in how students and faculty are associated with various factors. These differences appear across determinants such as Age, Education, College, Marital status, Residence, Gender, Tool Use, Motivation, Digital Transformation [DT] Training, PC Use, and Confidence, as well as digital competence domains including SDC, PU, EOU, ATU, BI, FC, and DTR.

**Figure 7:**
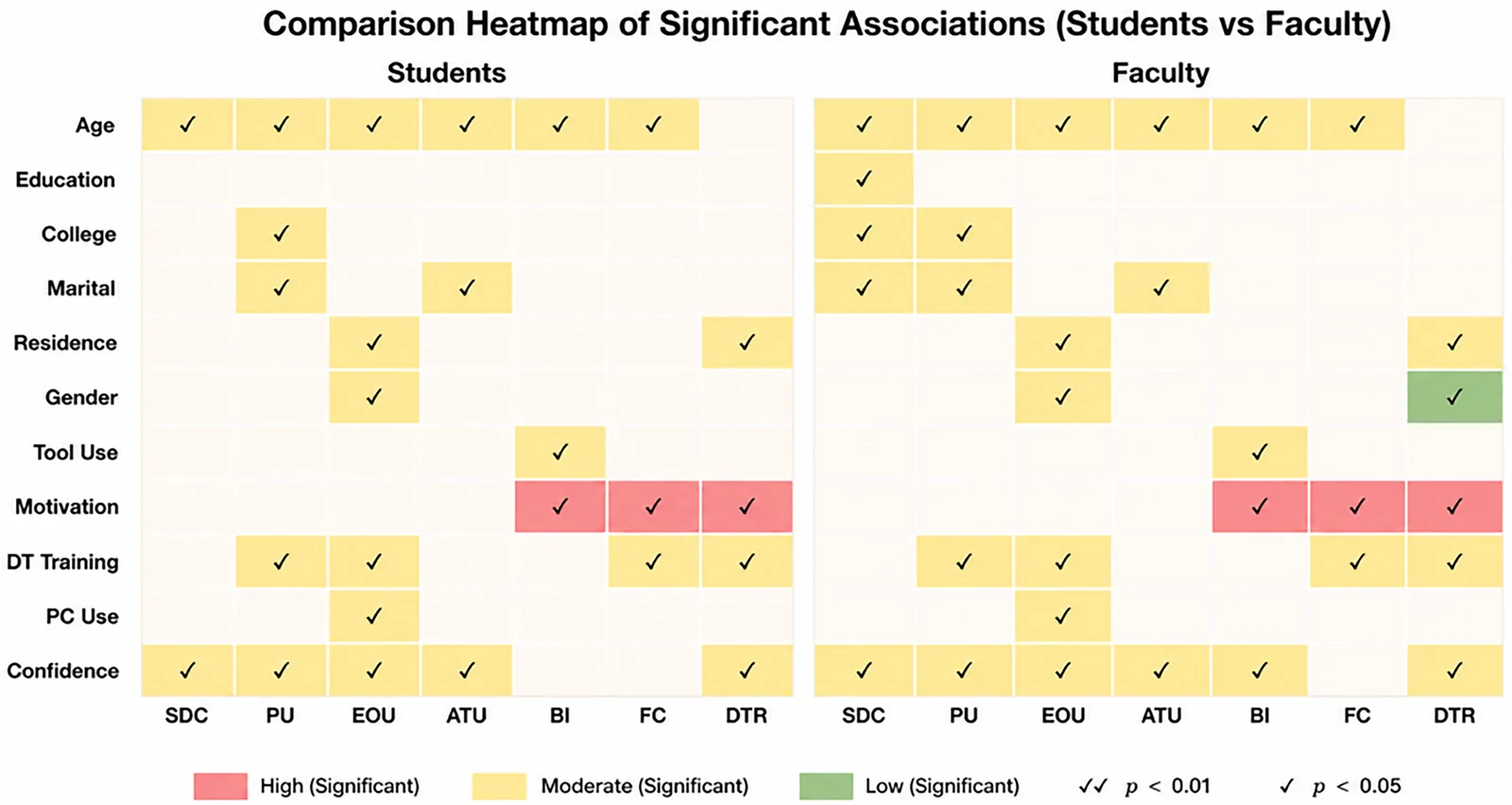
Combined comparison heatmaps (students vs. Faculty). Cells marked with a check (✓) indicate statistically significant relationships (*p* < 0.05).

Among students, associations are more focused and grouped. Strong links are observed with Age, Confidence, Motivation, and DT Training, suggesting that students are more affected by their readiness and training experiences. Motivation and Confidence relate to several competence areas, indicating that these internal factors play a key role in how students perceive the usefulness of digital learning, form intentions, and feel prepared for it.

For faculty, the heatmap shows a wider, more spread-out pattern, whereas the student heatmap highlights fewer factors. Faculty show significant associations with Age, Education, Motivation, and Confidence, as well as some influence from DT Training and Tool Use. This means faculty digital competence depends on a broader mix of demographic and professional factors, not just psychological ones. Age and Confidence stand out as consistently important across most areas, pointing to a stable competence structure linked to experience.

In summary, students’ digital competence is more affected by training and motivation, making it more responsive to interventions. Faculty digital competence, on the other hand, is more deeply rooted in demographic and professional experience. These differences suggest that digital transformation strategies should engage and train students, while supporting ongoing professional development and targeted upskilling for faculty.

 Table 10 shows the correlation matrix for digital competence, Technology Acceptance Model (TAM) constructs, and digital transformation readiness among students. The results reveal a consistent pattern of significant positive correlations across all variables (*p* < 0.01), showing that competence, technology acceptance, and readiness for digital transformation are closely linked.

**Table 10:** Correlation between students’ tools.

Variable/domain	Digital Competence		TAM										Digital Transformation Readiness	
			Perceived Usefulness		Perceived Ease of Use		Attitude Toward Use		Behavioral Intention to Use		Facilitating Conditions			
	r	p	r	p	r	p	r	p	r	p	r	p	r	p
Digital Competence			0.172	<0.001**	0.178	<0.001**	0.188	<0.001**	0.153	0.003	0.190	<0.001**	0.193	<0.001**
Perceived Usefulness	0.172	<0.001**			0.864	<0.001**	0.909	<0.001**	0.895	<0.001**	0.865	<0.001**	0.769	<0.001**
Perceived Ease of Use	0.178	<0.001**	0.864	<0.001**			0.887	<0.001**	0.881	<0.001**	0.858	<0.001**	0.750	<0.001**
Attitude Toward Use	0.188	<0.001**	0.909	<0.001**	0.887	<0.001**			0.916	<0.001**	0.873	<0.001**	0.773	<0.001**
Behavioral Intention to Use	0.153	0.003	0.895	<0.001**	0.881	<0.001**	0.916	<0.001**			0.905	<0.001**	0.794	<0.001**
Facilitating Conditions	0.190	<0.001**	0.865	<0.001**	0.858	<0.001**	0.873	<0.001**	0.905	<0.001**			0.773	<0.001**
Digital Transformation Readiness	0.193	<0.001**	0.769	<0.001**	0.750	<0.001**	0.773	<0.001**	0.794	<0.001**	0.773	<0.001**		

*Statistically significant at *p* < 0.05.

**Statistically significant at *p* < 0.01.

Digital competence had weak but significant positive links with all TAM dimensions and digital transformation readiness. These links included perceived usefulness (r = 0.172, *p* < 0.001), perceived ease of use (r = 0.178, *p* < 0.001), attitude toward use (r = 0.188, *p* < 0.001), behavioral intention to use (r = 0.153, *p* = 0.003), facilitating conditions (r = 0.190, *p* < 0.001), and overall digital transformation readiness (r = 0.193, *p* < 0.001). The results show that students’ digital competence is positively related to technology acceptance, but the small effect sizes indicate a limited contribution to readiness.

In contrast, the TAM constructs showed very strong interrelationships, with correlation coefficients ranging from r = 0.750 to r = 0.916 (all *p* < 0.001). Attitude toward use had the strongest association with behavioral intention to use (r = 0.916, *p* < 0.001), while perceived usefulness and perceived ease of use were also highly correlated with attitude toward use (r = 0.909 and r = 0.887, respectively), reinforcing the core TAM assumption that perceived utility and ease of use shape users’ attitudes and intentions toward technology adoption.

Facilitating conditions also showed strong positive correlations with all TAM components, particularly behavioral intention (r = 0.905, *p* < 0.001), indicating that institutional and environmental support play a critical role in students’ willingness to adopt digital tools.

Digital transformation readiness was significantly associated with all variables studied. The strongest connection was with behavioral intention to use (r = 0.794, *p* < 0.001), followed by attitude toward use (r = 0.773, *p* < 0.001) and facilitating conditions (r = 0.773, *p* < 0.001). This means that readiness for digital transformation depends more on behavioral and contextual factors than on digital competence alone. Overall, these findings show a clear, consistent pattern that aligns with the Technology Acceptance Model. Perceptions of usefulness, ease of use, and facilitating conditions together shape students’ attitudes, intentions, and readiness for digital transformation.

 Table 11 presents the correlations among digital competence, TAM constructs, and digital transformation readiness among faculty members. The results reveal statistically significant positive relationships. These relationships are mostly moderate among faculty but stronger among students, showing that faculty variables are more distinct and less closely connected.

**Table 11:** Correlations among faculty members’ study variables.

Variable	Digital Competence		TAM										Digital Transformation Readiness	
			Perceived Usefulness		Perceived Ease of Use		Attitude Toward Use		Behavioral Intention to Use		Facilitating Conditions			
	r	p	r	p	r	p	r	p	r	p	r	p	r	p
Digital Competence			0.048	0.369	0.121	0.024*	0.057	0.286	0.068	0.203	0.160	0.003*	0.102	0.057
Perceived Usefulness	0.048	0.369			0.354	<0.001**	0.372	<0.001**	0.411	<0.001**	0.257	<0.001**	0.471	<0.001**
Perceived Ease of Use	0.121	0.024*	0.354	<0.001**			0.358	<0.001**	0.317	<0.001**	0.312	<0.001**	0.398	<0.001**
Attitude Toward Use	0.057	0.286	0.372	<0.001**	0.358	<0.001**			0.384	<0.001**	0.303	<0.001**	0.462	<0.001**
Behavioral Intention to Use	0.068	0.203	0.411	<0.001**	0.317	<0.001**	0.384	<0.001**			0.344	<0.001**	0.506	<0.001**
Facilitating Conditions	0.160	0.003*	0.257	<0.001**	0.312	<0.001**	0.303	<0.001**	0.344	<0.001**			0.467	<0.001**
Digital Transformation Readiness	0.102	0.057	0.471	<0.001**	0.398	<0.001**	0.462	<0.001**	0.506	<0.001**	0.467	<0.001**		

The structural model demonstrated an acceptable fit to the data. The model fit indices were χ²/df = 1.28, Comparative Fit Index (CFI) = 0.999, Tucker–Lewis Index (TLI) = 0.997, Root Mean Square Error of Approximation (RMSEA) = 0.029, and Standardized Root Mean Square Residual (SRMR) = 0.008. These fit indices indicate that the proposed Technology Acceptance Model (TAM)-based structural model fits the observed relationships among the study variables.

r, correlation coefficient; TAM, Technology Acceptance Model.

*p < 0.05.

**p < 0.01.

Digital competence showed weak, mostly non-significant correlations with most TAM constructs. For example, perceived usefulness (r = 0.048, *p* = 0.369), attitude toward use (r = 0.057, *p* = 0.286), and behavioral intention to use (r = 0.068, *p* = 0.203) were all low. There was a small but significant link with perceived ease of use (r = 0.121, *p* = 0.024) and facilitating conditions (r = 0.160, *p* = 0.003), indicating that faculty digital competence is more related to ease of use and institutional support than to attitudes or behaviors.

In contrast, the TAM constructs showed consistent, moderate, and statistically significant relationships with one another. Perceived usefulness was positively linked to perceived ease of use (r = 0.354, *p* < 0.001), attitude toward use (r = 0.372, *p* < 0.001), behavioral intention (r = 0.411, *p* < 0.001), and facilitating conditions (r = 0.257, *p* < 0.001). Perceived ease of use was also significantly related to attitude toward use (r = 0.358, *p* < 0.001), behavioral intention (r = 0.317, *p* < 0.001), and facilitating conditions (r = 0.312, *p* < 0.001). These findings show that TAM constructs remain closely connected among faculty, but the links are weaker than among students.

Attitude toward use was significantly related to behavioral intention (r = 0.384, *p* < 0.001), supporting the TAM, which posits that attitudes influence behavior. Facilitating conditions were also significantly linked to all TAM variables, including behavioral intention (r = 0.344, *p* < 0.001). This shows that organizational and structural support play a key role in faculty use of digital tools.

Digital transformation readiness among faculty was moderately and significantly related to all TAM constructs. The strongest links were with behavioral intention to use (r = 0.506, *p* < 0.001), attitude toward use (r = 0.462, *p* < 0.001), and facilitating conditions (r = 0.467, *p* < 0.001). This suggests that behavioral motivation and institutional support are the main drivers of readiness, rather than digital competence alone. Constructs are meaningful predictors of faculty readiness for digital transformation. However, their interrelationships are less intense than those among students. This means faculty adoption behavior is influenced by a broader, more balanced mix of perceived usefulness, organizational context, and behavioral intention, rather than being primarily driven by digital competence.

## Analysis of moment structures (AMOS)-based structural model

To study how different factors affect digital transformation, we used a covariance-based structural equation modeling (CB-SEM) approach in IBM SPSS AMOS. Figure 8 represents the model which was based on the (TAM) and included psychological readiness (confidence and motivation), behavioral exposure (PC or laptop use), and organizational support (digital transformation training) as factors that influence key TAM elements and the intention to use digital technologies. The results show clear links between socio-demographic and digital-use factors and the main components of TAM. Overall, the model provides moderate support for TAM, with several significant connections aligning with our expectations.

**Figure 8:**
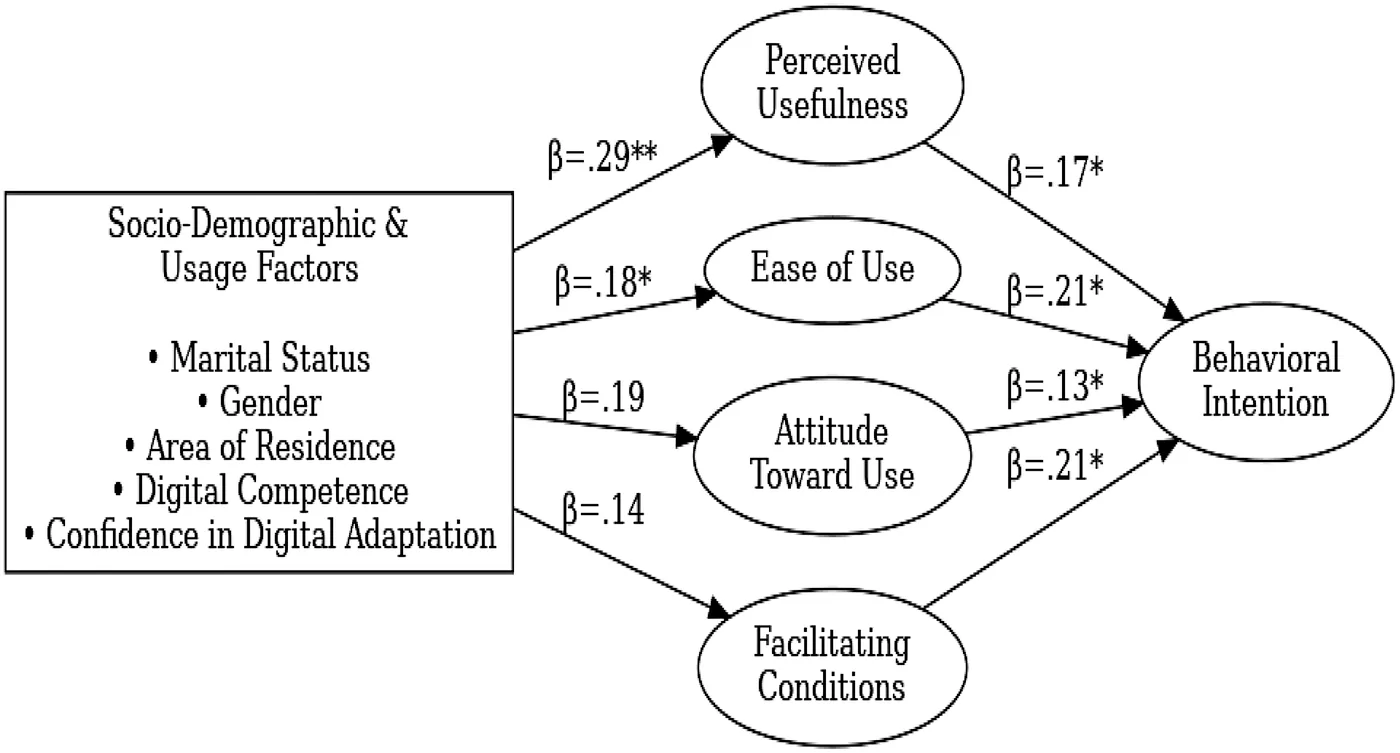
Structural model illustrating the relationships between socio-demographic and digital usage factors and technology acceptance model (TAM) constructs among faculty members and students. Standardized path coefficients are shown. Significant paths are indicated by *p < 0.05 and **p < 0.01. The overall model demonstrated acceptable fit (*χ*²/df = 1.28, CFI = 0.999, TLI = 0.997, RMSEA = 0.029, and SRMR = 0.008).

Considering the external factors, the combined set of socio-demographic and usage characteristics influenced parts of TAM in different ways. The biggest impact was on perceived usefulness (*β* = 0.29, *p* < 0.01), showing that background and digital readiness strongly shape people’s perception of the value of digital technologies. There was also a smaller but still important effect on ease of use (*β* = 0.18, *p* < 0.05), showing that these factors help shape how easy people perceive the technology to be.

On the other hand, the effects on attitude toward use (*β* = 0.19) and supporting conditions (*β* = 0.14) were weaker. This means that feelings about using technology and the sense of support from infrastructure are less directly influenced by background and usage factors. It suggests that attitudes are shaped more by thinking and beliefs than by demographics.

Within the TAM model, the results matched the theory’s predictions. Perceived usefulness (*β* = 0.17, *p* < 0.05) and ease of use (*β* = 0.21, *p* < 0.05) both had clear positive effects on intention to use technology, underscoring their importance for adoption. Support from the organization and technology (*β* = 0.21, *p* < 0.05) was also important, highlighting its role in digital engagement.

Although attitude toward use (*β* = 0.13, *p* < 0.05) was associated with intention to use technology, the small effect size suggests it accounts for only part of the relationship. Instead, behavioral intention is influenced more by direct thoughts about usefulness and ease of use than by feelings alone. Overall, the model shows that socio-demographic and usage factors affect behavioral intention indirectly through the TAM elements. The effect sizes are small to moderate, indicating that the model performs well in theory. To sharpen its implications, add factors such as digital self-efficacy, organizational culture, or perceived risk.

Because the study aimed to compare faculty members and students, the structural relationships were examined separately for each group. The findings demonstrated that the strength of associations among digital transformation readiness factors and TAM constructs varied between faculty and students, supporting the presence of group-specific patterns in technology acceptance and readiness for digital transformation. For the multi-group analysis, faculty and student groups were examined separately and then compared using a constrained structural-path model. The unconstrained model was compared with a model in which structural paths were constrained to be equal across groups. The chi-square difference test indicated a significant difference between the unconstrained and constrained models, *Δχ*² (8) = 17.64, *p* = 0.024, suggesting that some structural paths differed between faculty and students, despite the overall acceptable model fit.

 Table 12 compares the main factors predicting digital competence and examines TAM elements alongside faculty and student readiness for digital transformation. The results show both shared and differing factors across groups, highlighting distinct ways each group experiences digital transformation.

**Table 12:** Comparison of significant predictors of digital competence, TAM constructs, and digital transformation readiness among faculty (*n* = 350) and students (*n* = 377).

Outcome	Predictor	Faculty		Students	
		(*β*)	(*p*-value)	(*β*)	(*p*-value)
Digital competence	Confidence	0.189	<0.001***	0.190	<0.001***
	Motivation	—	—	0.296	<0.001***
	Marital status	—	—	0.115	0.016*
Perceived usefulness	Confidence	0.112	0.038*	0.050	0.013*
Ease of use	PC use	0.092	0.048*	—	—
	Confidence	—	—	0.139	0.020*
Attitude toward use	Digital competence	—	—	0.093	0.041*
	Confidence	—	—	0.146	0.015*
Behavioral intention	Digital competence	—	—	0.089	0.015*
	Confidence	—	—	0.124	0.039*
Facilitating conditions	DT training	0.121	0.025*	—	—
	Motivation	—	—	0.139	0.007*
Digital transformation readiness	DT training	0.112	0.037*	—	—
	Confidence	0.110	0.031*	—	—
	Motivation	0.105	0.050*	0.154	0.006*

*p < 0.05.

***p < 0.001.

For both groups, confidence in adapting to digital tools was the strongest and most reliable predictor. It significantly affected digital competence for faculty (*β* = 0.189, *p* < 0.001) and students (*β* = 0.190, *p* < 0.001), as well as perceptions of technology’s usefulness and readiness for digital transformation. For students specifically, it also influenced perceived ease of use, attitude, and intention to use technology, showing that digital self-efficacy is central to both thinking about and using technology.

Motivation had a stronger and more consistent effect among students than among faculty. For students, it was the most powerful predictor of digital competence (*β* = 0.296, *p* < 0.001) and also significantly influenced facilitating conditions and digital transformation readiness. Among faculty, motivation contributed only marginally to readiness (*β* = 0.105, *p* = 0.050), suggesting that intrinsic motivation is more critical for students’ digital engagement.

Among faculty, digital transformation training was associated with more support (*β* = 0.121, *p* = 0.025) and greater readiness (*β* = 0.112, *p* = 0.037), underscoring the importance of institutional support and professional development. For students, training did not matter, indicating that their digital readiness depends less on formal institutional support.

Behavioral and usage factors affected each group differently. For faculty, using a PC or laptop made technology easier to use (*β* = 0.092, *p* = 0.048). For students, confidence most influenced views on technology’s usability (*β* = 0.139, *p* = 0.020), and digital competence strongly shaped both attitudes toward technology and intentions to use it. This shows that skills are a key link to adopting new technology, especially for students.

Socio-demographic factors had little impact. Marital status had a small effect on students’ digital competence, but not on faculty, suggesting that psychological and organizational factors matter more than demographics for digital transformation.

 Table 13 summarizes the hypothesis testing results and provides evidence supporting the proposed model of digital transformation readiness among faculty members and students. Digital competence (knowledge) plays a fundamental role in shaping attitudes toward digital transformation, as shown by significant positive path coefficients for students (*β* = 0.29, *p* < 0.01) and faculty members (*β* = 0.31, *p* < 0.001). Individuals with higher digital knowledge are more likely to develop favorable perceptions and acceptance of digital transformation initiatives.

**Table 13:** Summary of hypothesis testing results

Hypothesis	Relationship Tested	Key Findings	Statistical Test	Main Evidence	Decision
H1	Knowledge has a significant positive effect on attitudes toward digital transformation.	Digital competence (knowledge) significantly predicted attitudes toward digital transformation among students and faculty members.	Structural Model Path Analysis	Students: β = 0.29, *p* < 0.01; Faculty: β = 0.31, *p* < 0.001	Supported
H2	Attitudes have a significant positive effect on readiness for digital transformation.	Positive attitudes toward digital transformation significantly enhanced readiness among both students and faculty members.	Structural Model Path Analysis	Students: β = 0.15, *p* < 0.01; Faculty: β = 0.18, *p* < 0.05	Supported
H3	Knowledge has a direct positive effect on readiness for digital transformation.	Digital competence (knowledge) demonstrated a significant direct effect on readiness among both groups.	Structural Model Path Analysis	Students: β = 0.21, *p* < 0.05; Faculty: β = 0.19, *p* < 0.05	Supported
H4	Attitudes mediate the relationship between knowledge and readiness for digital transformation.	Significant indirect effects of knowledge on readiness through attitudes were observed in both groups, indicating partial mediation.	Mediation Analysis (Indirect Effects)	Students: Indirect β = 0.0435; Faculty: Indirect β = 0.0558	Supported
H5	There are significant differences between faculty and students in the structural relationships among knowledge, attitudes, and readiness.	Differences were observed in the magnitude of several structural paths between faculty and students; however, the overall conceptual model was supported in both groups.	Comparative Structural Analysis	Variations in path coefficients across groups	Partially Supported

Attitudes toward digital transformation significantly influence readiness, indicating that positive perceptions of digital technologies enhance willingness and preparedness to engage in digital transformation processes. Although the effect sizes were modest, the relationships remained statistically significant for both students and faculty members.

In addition to its indirect influence through attitudes, digital competence had a significant direct effect on readiness, highlighting its dual role as both a cognitive resource and a practical determinant of preparedness for digital transformation. These findings emphasize that improving digital skills and competencies can directly strengthen readiness while simultaneously fostering more positive attitudes toward digital change.

The mediation analysis confirmed that attitudes partially mediate the relationship between knowledge and readiness, indicating that digital competence enhances readiness both directly and indirectly by fostering favorable attitudes. The presence of significant indirect effects in both groups supports theoretical models suggesting that attitudes serve as an important psychological mechanism linking knowledge to behavioral outcomes.

Regarding group comparisons, the results showed differences in the strength of structural relationships between faculty members and students, although the overall conceptual model remained valid across both populations. These variations suggest that faculty and students may experience digital transformation differently due to differences in professional responsibilities, educational experiences, exposure to technology, and organizational expectations. Therefore, while a common framework can be applied to both groups, tailored interventions may be necessary to address the specific needs of each population.

Overall, the findings support the proposed theoretical framework and underscore the importance of enhancing digital competence and fostering positive attitudes to improve readiness for digital transformation within higher education institutions.

## Discussion

This study evaluated five hypotheses based on the Technology Acceptance Model (TAM) and associated theories of technology adoption. The findings confirmed all five of the hypotheses(H). Digital competence positively impacted attitudes towards digital transformation (H1) and directly improved readiness (H 3). Additionally, attitudes significantly enhanced readiness (H 2). Moreover, attitudes partially mediated the relationship between digital competence and readiness (H 4). The multigroup analysis indicated variations between faculty members and students regarding the strength of these relationships and the factors affecting readiness (H 5), underscoring the need for tailored strategies for different groups to advance digital transformation in health professions education.

This study offers significant insights into digital transformation within health professions education by examining the digital skills, technology acceptance, and preparedness of faculty and students at KSAU-HS. The results suggest that readiness for digital transformation encompasses more than just access to technology; it is also shaped by psychological, technological, and organizational factors, including perceived usefulness, ease of use, facilitating conditions, confidence, and institutional support. These outcomes are consistent with the current literature, which highlights that effective digital transformation in health professions education necessitates both individual competence and organizational preparedness. This is especially crucial as digital technologies are increasingly incorporated into teaching, clinical training, telehealth, artificial intelligence, and data-driven healthcare practices (32–35).

The SEM analysis provided strong evidence for the (TAM), revealing that perceived usefulness, perceived ease of use, and facilitating conditions significantly impacted the intention to adopt technology. These findings align with earlier research, indicating that both individual and organizational elements influence technology acceptance through TAM constructs. For instance, Mastour et al. identified perceived usefulness and ease of use as key factors in technology acceptance among faculty and students. Similarly, Lee et al. (36) emphasized TAM’s ongoing relevance for understanding technology adoption in health professions education, particularly regarding institutional support and learner characteristics (36, 37). The current results underscore the necessity of developing user-friendly digital environments backed by sufficient institutional resources.

Faculty members exhibited a notably higher level of digital preparedness, perceived usefulness, perceived ease of use, positive attitudes, and intention to use technology than students. This disparity might be attributed to their increased interaction with institutional digital platforms, online teaching systems, academic management tools, and professional development opportunities. Similar conclusions have been drawn in research indicating that healthcare educators’ digital readiness is bolstered by their experience, confidence in using technology, and the alignment of technology with their professional duties (21, 36). However, some research has indicated that younger learners often display greater familiarity with technology than faculty members (37). Nevertheless, the current findings imply that being accustomed to everyday digital technologies does not automatically equate to readiness for academic, professional, or clinical contexts. Although students reported frequent use of technology, the considerable variation in their readiness and confidence suggests that mere exposure to technology may not be sufficient to cultivate professional digital competence.

Confidence in adapting to digital environments emerged as a key predictor for both student groups. It played a significant role in forecasting digital competence among faculty and students and in shaping students’ perceptions of usefulness, ease of use, attitudes, and intentions to engage. These results align with growing evidence highlighting digital self-efficacy as a crucial factor in successful digital transformation. Sun and Yoon demonstrated that digital self-efficacy mediates the relationship between digital transformation efforts and educational outcomes, emphasizing that infrastructure alone is insufficient for successful adoption (21). Likewise, research in medical education has underscored the role of digital competence in facilitating self-directed learning, engagement, and effective use of digital information resources (38). Consequently, confidence should be viewed as a changeable outcome that can be enhanced through training, practice, and supportive learning environments.

Motivation was identified as the most significant predictor of students’ digital competence, greatly impacting both facilitating conditions and their preparedness for online learning. This aligns with existing research highlighting the critical roles of engagement, relevance, and self-directed learning in digital education settings (38, 39). According to Kröplin et al., involvement in digital health education activities enhances students’ perception of the importance of digital health and boosts their confidence in using digital technologies (40). Conversely, motivation had a relatively minor effect on faculty readiness, suggesting that organizational expectations, workload, professional duties, and institutional support may play a more substantial role in faculty adoption of AI tools.

The finding that formal digital transformation training improved facilitating conditions and readiness among faculty further highlights the importance of structured professional development (46). Previous European studies have similarly reported fragmented digital health training among healthcare professionals and emphasized the need for systematic educational initiatives to address competency gaps (41, 42). While faculty appear to benefit from dedicated training programs, students may develop digital competence more effectively through curriculum-integrated learning experiences, including simulations, digital case studies, telehealth applications, artificial intelligence tools, and other authentic learning activities, underscoring the value of integrating digital learning throughout the curriculum.

Another significant discovery was the restricted impact of socio-demographic traits on preparedness for digital transformation. In addition to a slight link between marital status and student digital skills, demographic factors were typically poor indicators. This indicates that changeable psychological and organizational factors may have a stronger impact on preparedness for digital transformation than unchangeable personal traits. While international research has highlighted links among age, education, prior digital exposure, and digital skills (43, 44), the relatively uniform academic setting and common technological framework at KSAU-HS might account for the limited demographic influences observed in this study.

Correlation analyses provide further insight into the disparities between faculty and students. Within the faculty, digital competence was primarily linked to perceived ease of use and supportive conditions, indicating that technology adoption is heavily influenced by practical factors such as usability and task efficiency. These results align with the task-technology fit theory, which suggests that technology is more likely to be adopted when it effectively supports users in fulfilling their professional duties and goals (21). Consequently, faculty development programs must focus on discipline-specific uses and practical incorporation into teaching and evaluation practices.

Conversely, students demonstrated more robust connections among TAM constructs, suggesting that technology adoption is more closely associated with attitudes, beliefs, and perceptions. Students who viewed digital technologies as beneficial and user-friendly exhibited more favorable attitudes, stronger behavioral intentions, and greater willingness to undergo digital transformation. Comparable results have been found in blended learning studies, highlighting the significance of digital literacy, perceived usefulness, and engagement in influencing students’ learning experiences (42). Nonetheless, research has indicated that students’ perceptions of digital learning can be affected by factors such as platform quality, workload, digital fatigue, and perceived relevance to clinical practice (20, 32). These results indicate that relying solely on technology use should not be seen as proof of readiness for a digital transformation.

While the suggested model accounted for considerable variation in technology acceptance and readiness, the small effect sizes observed for certain pathways suggest opportunities for further theoretical advancement. Future studies should include more constructs, such as digital self-efficacy, perceived risk, digital stress, organizational culture, data privacy issues, artificial intelligence readiness, and task–technology alignment. Recent findings indicate that modern digital transformation goes beyond conventional TAM assumptions and increasingly includes wider concerns related to ethics, data governance, workflow integration, and digital equity (45, 46). Combining TAM with additional frameworks, such as Unified Theory of Acceptance and Use of Technology (UTAUT), models of organizational readiness, and task–technology fit theory, could offer a broader perspective on digital transformation in health professions education.

The results have multiple practical implications for education in health professions. Initiatives aimed at faculty must emphasize structured professional growth, practical training, peer mentorship, and ongoing technical support to enable the effective integration of digital tools into teaching, evaluation, simulation, learning analytics, and (AI)-enhanced education. For learners, teaching methods should prioritize boosting motivation, confidence, and involvement while integrating digital health skills throughout the program. Recent evidence shows that structured digital health education enhances students’ confidence, competence, and understanding of digital technologies in future healthcare practices (47, 48).

## Strengths of the study

This study stands out for several reasons. First, it offers a thorough comparison of how digital transformation affects employees, using several analytical methods and including faculty and students in health professions education, an area that has not been widely studied.

Second, the study employs several analytical methods, including correlation analysis, multiple linear regression, and CB-SEM. This approach supports the findings with theory and data.

Third, the study is based on the (TAM). By adding components such as confidence, motivation, technology use, training, and support, the model explains the results more fully.

Fourth, the study identifies distinct patterns between faculty and students, highlighting the roles of student motivation and faculty organizational support. This comparative insight represents a key contribution to the literature and provides pragmatic implications for specific interventions.

Fifth, the study’s large, varied sample across several health fields makes the results more reliable and relevant to the institution.

## Limitations of the study

While this study has strengths, it also has some limitations. First, because it uses a cross-sectional design, we cannot determine cause-and-effect relationships between variables. The structural equation model shows possible directions, but only long-term studies can confirm cause and timing.

Second, the data were collected via self-report questionnaires, which can introduce response biases, such as social desirability bias or recall inaccuracies.

Third, the study took place at only one university (KSAU-HS), so the results may not generalize to other schools or healthcare education systems with different resources, cultures, or levels of digital technology experience.

Fourth, students were selected using stratified sampling. However, some participants were also chosen for convenience. This could mean that people more interested in digital technology were more likely to participate, potentially introducing selection bias.

Fifth, the SEM model explained only a moderate amount of the results. This means other factors—digital self-confidence, workplace culture, perceived risks, digital stress, or access to technology—were excluded but may help explain digital transformation readiness.

Finally, the study examined TAM factors but did not measure digital skills or technology use. Measuring these would provide a fuller picture of the results of digital transformation.

## Practical and theoretical implications

### Practical implications

The results offer numerous practical implications for universities, health profession educational organizations, and leaders in digital transformation:

- Institutions should develop comprehensive digital transformation plans that combine technological infrastructure, faculty development, student support, and ongoing evaluation systems. The significant impact of digital self-efficacy and motivation on preparedness indicates that digital transformation efforts must go beyond providing technology; they should also include strategies that enhance users’ confidence and involvement with digital tools.
- Institutions should offer continuous professional development for faculty members that emphasizes digital pedagogy, instructional design, and effective technology integration. Practical, hands-on training coupled with peer support networks can improve faculty confidence and preparedness to embrace innovative educational technologies. Educational programs for students should include motivation-boosting learning environments, hands-on learning experiences, simulation activities, telehealth resources, and AI-enhanced educational tools to improve digital skills and preparedness.
- At the organizational level, the results highlight the importance of enabling conditions such as technical assistance, intuitive platforms, and organizational commitment to digital ecosystems. Policymakers and educational leaders should align digital transformation efforts with national strategies for digital health and education while integrating digital competency frameworks into curricula, accreditation criteria, and workforce development programs.

### Theoretical implications

This research contributes to the expanding body of work on digital transformation preparedness in health professions education by extending the use of the (TAM) within higher education in the Kingdom of Saudi Arabia. In line with TAM, perceived ease of use and perceived usefulness emerged as key factors influencing behavioral intention, reinforcing the ongoing significance of technology acceptance theory in studies on digital transformation.

The results emphasize the significance of psychological and organizational readiness elements in addition to conventional demographic traits. The significant impact of confidence on digital adaptation and motivation indicates that preparedness for digital transformation is shaped by broader self-efficacy and readiness factors that complement the conventional TAM variables. Furthermore, the differing predictors identified between faculty and students suggest that readiness for digital transformation may operate differently across stakeholder groups, underscoring the need for context-aware models of technology adoption in health professions education.

By combining perspectives on readiness, technology acceptance, and organizational support, this study enhances theoretical insight into how digital transformation can be effectively implemented in educational institutions undergoing rapid technological change.

## Conclusion

Health professionals in higher education were studied to see how ready they are for using digital technologies and what factors shape their readiness. Results showed that psychological and organizational factors were more influential than demographic characteristics with regard to digital transformational readiness. Adapting to digital technology was the primary predictor of readiness among both faculty members and students, but motivation was particularly important for students. Faculty members needed to feel confident after receiving institution-supplied training and support in order to facilitate their ability to adapt and use digital technologies. Additionally, the results validated some elements of the Technology Acceptance Model, specifically perceived usefulness, perceived ease of use, and facilitating conditions were all found to contribute to the level of readiness for digital transformation in health professions education. In addition to contributing to the literature on digital transformation, this study has practical implications for successful implementation of digital transformation, based on individual and institutional factors. Therefore, educational organizations are encouraged to develop a holistic digital transformation strategy that includes (a) an appropriate technological infrastructure, (b) on-going technical support, (c) structured opportunities for training, and (d) supportive digital ecosystems. Establishment of monitoring and evaluation mechanisms to evaluate the readiness for digital transformation and the adoption of technology over time, will assist in developing continuous improvement and evidence-based decision-making.

Also, in relation to faculty development, the findings of this study suggest the need for sustained efforts in faculty development that emphasize hands-on training in digital pedagogy and instructional design and integration of technology; and the provision of peer mentoring and communities of practice to facilitate sharing of knowledge and innovation. In relation to students, educational organizations should develop an engaging and motivational learning environment for students that uses interactive, simulation-based, and case-based digital learning approaches. The inclusion of digital health competencies across the curriculum, as well as the provision of experiential learning opportunities (via telehealth simulation, AI applications, and other emerging technologies) will assist in preparing students for future practice in healthcare.

Digital transformation initiatives at the policy level must be aligned with national and institutional digital health strategies and integrated into curriculum planning and accreditation standards by means of establishing digital competence frameworks. Academic institutions are encouraged to collaborate with healthcare organizations to ensure that educational opportunities related to digital competency continue to meet the needs of the evolving clinical practice and workforce requirements.

Longitudinal, experimental, and multi-institutional designs should be used in future research to examine the causal relationships between digital healthcare technologies and evaluate the effectiveness of various interventions designed to improve digital readiness. Future research should examine additional variables such as digital self-efficacy, digital stress, organizational culture, and perceived risk, and should include considerate measures of digital competency and actual technology utilization in addition to cross-national comparative studies. This will improve the generalizability of the findings and allow for a comprehensive understanding of digital transformation readiness in health professions’ education.

## Data Availability

The raw data supporting the conclusions of this article will be made available by the authors, without undue reservation, upon reasonable request to the corresponding author.
