# Supplementary material for: Decoding digital transformation in health professions education: a comparative analysis of knowledge, attitudes, and readiness among faculty and students at KSAU-HS
Source: Front Digit Health. 2026 Sep 15;8:1881377. doi: 10.3389/fdgth.2026.1881377 (PMC13619956; doi:10.3389/fdgth.2026.1881377)
Supplement: Supplementary file 1 [file Datasheet1.pdf]

Appendix 1 Faculty survey

Demographic Data:

1. **Age: _________** years
2. **Gender:**
   - ☐ Male
   - ☐ Female
3. **Educational Attainment:**
   - ☐ Still studying for Bachelor’s
   - ☐ Bachelor’s
   - ☐ Master’s
   - ☐ Doctorate
   - ☐ Other (please specify): _________
4. **Marital Status:**
   - ☐ Single
   - ☐ Married
   - ☐ Divorced
   - ☐ Widowed
5. **Number of Offspring:**
   - ☐ None
   - ☐ 1
   - ☐ 2
   - ☐ 3
   - ☐ 4
   - ☐ 5+
6. **Area of Residence:**
   - ☐ Central Region
   - ☐ Western Region
   - ☐ Eastern Region
7. **KSAU College Affiliation:**
   - ☐ College of Medicine
   - ☐ College of NursingDentist
   - ☐ College of Dentistry
   - ☐ College of Pharmacy
   - ☐ College of Public health and health information
   - ☐ College of Applied Medical Sciences
   - ☐ College of Health profession
   - ☐ Other (please specify): _________
8. **Years of Teaching/Professional Experience: ______** years

9. **How often do you use digital tools (e.g., learning management systems, online research databases, digital communication platforms) for your academic or professional activities?**
    - ☐ Daily
    - ☐ Weekly
    - ☐ Monthly
    - ☐ Rarely
    - ☐ Never
10. **How would you rate your own digital competence (e.g., navigating software, using digital tools effectively)?**
    - ☐ Very low
    - ☐ Low
    - ☐ Moderate
    - ☐ High
    - ☐ Very high
11. **Which digital devices do you primarily use for academic/professional purposes? (Select all that apply)**
    - ☐ Laptop
    - ☐ Desktop computer
    - ☐ Smartphone
    - ☐ Tablet
    - ☐ None
12. **How reliable is your internet connection for conducting digital activities?**
    - ☐ Very unreliable
    - ☐ Somewhat unreliable
    - ☐ Neutral
    - ☐ Somewhat reliable
    - ☐ Very reliable

14. **On a scale of 1 to 5, how motivated are you to participate in digital transformation initiatives in your academic/professional environment?**

- ☐ 1 (Not at all motivated)
- ☐ 2
- ☐ 3
- ☐ 4
- ☐ 5 (Very motivated)

15. Have you received any formal training on digital transformation or technology integration in your academic or professional setting?

- ☐ Yes

- ☐ No
- **If yes, please specify the type of training: ________**
-

16. How confident are you in adapting to new digital tools and technologies?

- ☐ Not at all confident
- ☐ Slightly confident
- ☐ Moderately confident
- ☐ Very confident
- ☐ Extremely confident

- **Appendix 2**

Teacher's Digital Competence (SDC) Scale:

Please rate your competency level on using digital transformation in your teaching work:

No.	Statements	NC	SC	MC	C	HC
**1.**	"I create digital content for my lessons, such as interactive presentations, videos, or e-books."					
**2.**	"I am able to edit and adapt digital resources for various classroom activities."					
**3.**	"I use digital tools to enhance lesson content, making it engaging and accessible to students."					
**4.**	"I utilize digital platforms to foster collaboration among students (e.g., group projects, discussions)."					
**5.**	"I incorporate digital tools to create a more inclusive learning experience."					
**6.**	"I adapt my teaching strategies based on the digital tools available to maximize student engagement."					
**7.**	"I communicate with students through digital means, such as email, messaging apps, or virtual classrooms."					
**8.**	"I engage parents in their child's learning process using digital communication tools."					
**9.**	"I collaborate with colleagues on digital platforms to share teaching resources and insights."					
**10.**	"I can find reliable online resources that enhance my lesson plans."					
**11.**	"I critically evaluate digital content to ensure accuracy and relevance for my students."					
**12.**	"I teach students how to assess digital information sources for reliability and accuracy."					

**13.**	"I am knowledgeable about online safety and incorporate this knowledge into classroom practices."					
**14.**	"I use strategies to protect students' personal information during digital activities."					
**15.**	"I inform students about data privacy and responsible digital behavior."					
**16.**	"I use digital tools to assess student learning and keep track of their progress."					
**17.**	"I provide students with timely feedback using digital platforms."					
**18.**	"I adjust assessments to leverage the benefits of digital tools, enhancing student understanding."					
**19.**	"I seek out professional development opportunities focused on digital competence."					
**20.**	"I stay informed about new technologies that could benefit my teaching practice."					
**21.**	"I actively improve my digital skills to stay current in a rapidly changing educational environment."					

The Technology Acceptance Model (TAM) Scale:

Please rate your readiness to use digital transformation in education and organizational environments

No.	Statements	SA	agree	Neutral	Disagree	SD
1.	"Using digital tools improves the quality of my work."					
2.	"I find digital tools beneficial for my academic tasks."					
3.	"Digital tools enhance my productivity in academic settings."					
4.	"Using technology in my studies increases my effectiveness."					
5.	"Learning to operate new technologies is easy for me."					
6.	"I find it easy to navigate digital tools."					
7.	"My interaction with digital tools is clear and understandable."					
8.	"I feel confident using digital platforms without extensive training."					
9.	"I feel positive about using digital tools in my teaching/learning."					
10.	"Using technology in my studies is enjoyable."					

11.	"I prefer using digital tools over traditional methods."					
12.	"I would recommend using digital tools to my peers."					
13.	"I plan to use digital tools more often in my educational activities."					
14.	"I intend to incorporate digital technology into my future projects."					
15.	"I will actively seek opportunities to use digital tools in my learning."					
16.	"I am likely to try new digital technologies when available."					
17.	"My institution provides sufficient resources to support technology use."					
18.	"I have access to adequate training for using digital tools."					
19.	"Technical support is available when I face issues with technology."					
20.	"I have the necessary infrastructure (e.g., internet access) to use digital tools effectively."					

- **Appendix 5**

The Digital Transformation Readiness Scale for Education and Universities.

Please rate your acceptance and attitudes toward DT in Education and university environments.

No.	Statements	SA	agree	Neutral	Disagree	SD
1.	"I have the necessary skills to use digital tools effectively in my teaching/learning."					
2.	"I am comfortable learning new digital platforms and tools for academic use."					
3.	"I feel confident integrating digital tools into educational tasks."					
4.	Digital transformation will improve the quality of teaching and learning in universities."					
5.	"Digital tools make it easier to access and share educational resources."					
6.	"Implementing digital transformation will enhance collaboration among faculty and students."					
7.	"I am open to adapting my teaching/learning methods to incorporate digital tools."					

8.	"I am willing to embrace new digital methods for administrative or academic tasks."					
9.	"I believe adapting to digital transformation is essential for educational success."					
10	"I plan to use digital tools more extensively in my teaching/learning activities."					
11	"I intend to participate in training sessions related to digital transformation."					
12	"I am likely to use digital tools to collaborate with others in my institution."					
13	"My university provides adequate training resources for digital tools."					
14	"There is strong leadership support for digital transformation within my institution."					
15	"My university actively encourages the use of digital tools in education."					
16	"Our university provides the necessary digital infrastructure for online learning."					
17	"The resources available at my institution are sufficient to support digital transformation."					
18	"My university has reliable IT support to assist with digital tools and platforms."					
