# Supplementary material for: Decoding digital transformation in health professions education: a comparative analysis of knowledge, attitudes, and readiness among faculty and students at KSAU-HS
Source: Front Digit Health. 2026 Sep 15;8:1881377. doi: 10.3389/fdgth.2026.1881377 (PMC13619956; doi:10.3389/fdgth.2026.1881377)
Supplement: Supplementary file 2 [file Datasheet2.pdf]

Appendix 2 student

Demographic Data:

1. **Age: ________** years
2. **Gender:**
   - ☐ Male
   - ☐ Female
3. **Educational Attainment:**
   - ☐ Still studying for Bachelor’s
   - ☐ Bachelor’s
   - ☐ Master’s
   - ☐ Doctorate
   - ☐ Other (please specify): ________
4. **Marital Status:**
   - ☐ Single
   - ☐ Married
   - ☐ Divorced
   - ☐ Widowed
5. **Number of Offspring:**
   - ☐ None
   - ☐ 1
   - ☐ 2
   - ☐ 3
   - ☐ 4
   - ☐ 5+
6. **Area of Residence:**
   - ☐ Central Region
   - ☐ Western Region
   - ☐ Eastern Region
7. **KSAU College Affiliation:**
   - ☐ College of Medicine
   - ☐ College of NursingDentist
   - ☐ College of Dentistry
   - ☐ College of Pharmacy
   - ☐ College of Public health and health information
   - ☐ College of Applied Medical Sciences
   - ☐ College of Health profession
   - ☐ Other (please specify): ________
8. **Years of Teaching/Professional Experience: ______** years
9. **How often do you use digital tools (e.g., learning management systems, online research databases, digital communication platforms) for your academic or professional activities?**
   - ☐ Daily
   - ☐ Weekly
   - ☐ Monthly
   - ☐ Rarely
   - ☐ Never
10. **How would you rate your own digital competence (e.g., navigating software, using digital tools effectively)?**
    - ☐ Very low

- ☐ Low
- ☐ Moderate
- ☐ High
- ☐ Very high

11. **Which digital devices do you primarily use for academic/professional purposes? (Select all that apply)**
    - ☐ Laptop
    - ☐ Desktop computer
    - ☐ Smartphone
    - ☐ Tablet
    - ☐ None
12. **How reliable is your internet connection for conducting digital activities?**
    - ☐ Very unreliable
    - ☐ Somewhat unreliable
    - ☐ Neutral
    - ☐ Somewhat reliable
    - ☐ Very reliable

14. **On a scale of 1 to 5, how motivated are you to participate in digital transformation initiatives in your academic/professional environment?**

- ☐ 1 (Not at all motivated)
- ☐ 2
- ☐ 3
- ☐ 4
- ☐ 5 (Very motivated)

15. Have you received any formal training on digital transformation or technology integration in your academic or professional setting?

- ☐ Yes
- ☐ No
- **If yes, please specify the type of training: ________**
-

16. How confident are you in adapting to new digital tools and technologies?

- ☐ Not at all confident
- ☐ Slightly confident
- ☐ Moderately confident
- ☐ Very confident
- ☐ Extremely confident

Students' Digital Competence (SDC) Scale:

Please rate your competency level on using digital transformation in your learning experiences both theory and practical :

No.	Statements	NC	SC	MC	C	HC
**1.**	"I am comfortable using digital devices (e.g., computers, tablets) for learning and personal activities."					
**2.**	"I know how to perform basic functions on digital devices (e.g., saving files, creating folders)."					
**3.**	"I am able to type efficiently and use shortcuts in word processing software."					
**4.**	"I know how to manage files on digital devices (e.g., download, upload, delete files)."					
**5.**	"I can use presentation software (e.g., PowerPoint, Google Slides) to create visual aids for assignments."					
**6.**	"I am able to search for information online effectively (e.g., using keywords, filtering results)."					
**7.**	"I know how to evaluate the reliability and accuracy of information from online sources."					
**8.**	"I understand the importance of citing digital sources when using information for assignments."					
**9.**	"I can distinguish between trustworthy and unreliable websites and sources."					
**10.**	"I know how to organize digital information and resources for efficient study and research."					
**11.**	"I use digital platforms to collaborate with classmates on group projects (e.g., Google Docs, online forums)."					
**12.**	"I can communicate effectively through email and instant messaging apps (e.g., Gmail, WhatsApp)."					
**13.**	"I know proper online etiquette for communicating with teachers and peers."					
**14.**	"I participate in online discussions respectfully and constructively."					
**15.**	"I am familiar with video conferencing tools (e.g., Zoom, Microsoft Teams) and can use them for virtual meetings."					
**16.**	"I can troubleshoot basic issues on digital devices (e.g., internet connectivity, software updates)."					
**17.**	"I am able to adjust settings on digital devices (e.g., adjusting sound, connecting to Wi-Fi)."					
**18.**	"I can find and use help resources (e.g., online FAQs, tutorials) to solve digital problems."					
**19.**	"I am comfortable learning to use new digital tools or apps when needed."					
**20.**	"I can adapt to new digital platforms and technologies as they are introduced."					

**21.**	"I understand the importance of protecting my personal information online (e.g., using strong passwords)."					
**22.**	"I am aware of privacy settings on social media and other platforms and know how to use them."					
**23.**	"I know how to recognize and avoid online threats, such as phishing and malware."					
**24.**	"I understand what constitutes appropriate and respectful behavior online."					
**25.**	"I know how to manage my digital footprint and understand the impact of my online actions."					

The Technology Acceptance Model (TAM) Scale:

Please rate your readiness to use digital transformation in education and organizational environments

No.	Statements	SA	agree	Neutral	Disagree	SD
1.	"Using digital tools improves the quality of my work."					
2.	"I find digital tools beneficial for my academic tasks."					
3.	"Digital tools enhance my productivity in academic settings."					
4.	"Using technology in my studies increases my effectiveness."					
5.	"Learning to operate new technologies is easy for me."					
6.	"I find it easy to navigate digital tools."					
7.	"My interaction with digital tools is clear and understandable."					
8.	"I feel confident using digital platforms without extensive training."					
9.	"I feel positive about using digital tools in my teaching/learning."					
10.	"Using technology in my studies is enjoyable."					
11.	"I prefer using digital tools over traditional methods."					
12.	"I would recommend using digital tools to my peers."					
13.	"I plan to use digital tools more often in my educational activities."					
14.	"I intend to incorporate digital technology into my future projects."					
15.	"I will actively seek opportunities to use digital tools in my learning."					
16.	"I am likely to try new digital technologies when available."					
17.	"My institution provides sufficient resources to support technology use."					
18.	"I have access to adequate training for using digital tools."					
19.	"Technical support is available when I face issues with technology."					
20.	"I have the necessary infrastructure (e.g., internet access) to use digital tools effectively."					

The Digital Transformation Readiness Scale for Education and Universities.

Please rate your acceptance and attitudes toward DT in Education and university environments.

No.	Statements	SA	agree	Neutral	Disagree	SD
1.	"I have the necessary skills to use digital tools effectively in my teaching/learning."					
2.	"I am comfortable learning new digital platforms and tools for academic use."					
3.	"I feel confident integrating digital tools into educational tasks."					
4.	Digital transformation will improve the quality of teaching and learning in universities."					
5.	"Digital tools make it easier to access and share educational resources."					
6.	"Implementing digital transformation will enhance collaboration among faculty and students."					
7.	"I am open to adapting my teaching/learning methods to incorporate digital tools."					
8.	"I am willing to embrace new digital methods for administrative or academic tasks."					
9.	"I believe adapting to digital transformation is essential for educational success."					
10	"I plan to use digital tools more extensively in my teaching/learning activities."					
11	"I intend to participate in training sessions related to digital transformation."					
12	"I am likely to use digital tools to collaborate with others in my institution."					
13	"My university provides adequate training resources for digital tools."					
14	"There is strong leadership support for digital transformation within my institution."					
15	"My university actively encourages the use of digital tools in education."					
16	"Our university provides the necessary digital infrastructure for online learning."					
17	"The resources available at my institution are sufficient to support digital transformation."					
18	"My university has reliable IT support to assist with digital tools and platforms."					
